# Needs and barriers of healthcare teams to implement real-time AI & predictive analytics in cardiothoracic ICU: a field study

**DOI:** 10.3389/fdgth.2026.1943663

**Published:** 2026-10-30

**Authors:** Carey L. Barry, Sydney K. Purdue, Mahsan Nourani, Clifton Forlines, Matthew S. Goodwin, Melanie Tory

**Affiliations:** 1 Bouvé College of Health Sciences, Northeastern University, Boston, MA, United States; 2 Khoury College of Computer Sciences, Northeastern University, Boston, MA, United States; 3 College of Arts, Media and Design, Northeastern University, Boston, MA, United States; 4 The Roux Institute, Northeastern University, Portland, ME, United States; 5 Khoury College of Computer Sciences, Northeastern University, Oakland, CA, United States

**Keywords:** AI user interface design, artificial intelligence (AI) development framework, cardiothoracic intensive care unit (CT-ICU), field study, healthcare, human-computer interaction (HCI), predictive analytics, technostress

## Abstract

**Introduction:**

Artificial Intelligence (AI) offers significant opportunities to improve healthcare quality, especially in critical care settings, where timely decisions are essential yet challenging due to the volume of continuously accumulating patient data. AI algorithms can analyze data in real time, summarize key information for busy care teams, and alert clinicians to critical events and important care milestones. However, successful implementation requires first understanding the needs and barriers care teams face, including needs that are currently missing from their workflow, and what obstacles to integration exist.

**Methods:**

To address these questions, we conducted a field study in a Cardiothoracic Intensive Care Unit (CT-ICU) to evaluate user needs for implementing real-time AI predictive analytics. We observed and interviewed fourteen participants (*n* = 14), including nursing staff, advanced practice providers (NP/PAs), cardiovascular critical care attending physicians, and surgeons. Through a qualitative thematic analysis, we identified key considerations for developing and implementing static and real-time AI and predictive tools in the CT-ICU, aligning with the Technology Acceptance Model (TAM) constructs of *perceived usefulness* and *perceived ease of use*.

**Findings:**

Key themes included: (1) the system must have *added clinical value* to the clinical team and patients, (2) *trust in the AI system* should be prioritized through co-design processes, transparency in the system, and consistent prediction accuracy, and (3) *integration into workflow* was critical, including robust onboarding and minimizing alarm fatigue, charting burden, click burden, and technology burden with consideration of personal wellness.

**Conclusion:**

As a synthesis of our findings, we identify a series of key design insights and suggest a preliminary framework for AI technology development focused on clinical team trust and adoption, the *Trusted AI for Clinical Teams (TACT)* framework. These findings offer the potential to bridge from AI research to clinical implementation.

## 1 Introduction

Cardiothoracic Intensive Care Units (CT-ICUs) focus on caring for cardiothoracic surgical patients under the care of a highly trained, specialized care team (1, 2). Morbidity and mortality among critically ill patients remain high, with known variation between high-volume and lower-volume centers (3–10). Higher volume centers have been shown to have better outcomes across both ICU and CT surgical settings (7, 10). Variation in outcomes may be associated with failure-to-rescue events (9), defined as preventable adverse outcomes resulting from complications of an underlying illness (11, 12). Early recognition has been identified as a critical factor in reducing failure-to-rescue events and, thus, morbidity and mortality (12, 13). As such, there is a substantial opportunity for predictive artificial intelligence (AI) algorithms to reduce mortality in the CT-ICU by enabling early identification and intervention.

In this paper, we report on a field study that we conducted at an academic medical center CT-ICU to evaluate the needs and barriers to implementing static and real-time AI in a previously unstudied critical care setting (CT-ICU) with highly complex and specialized needs. Static predictions are batch or point-in-time predictions based on collected data, compared to real-time predictions, which make continuous or serial predictions based on real-time changes in patient data (14, 15). The focus was on informing the development of an effective user interface that enables healthcare providers to visualize, interpret, and apply data from an AI algorithm predicting future adverse outcomes for individual patients. In particular, the proposed algorithm would assign patient-specific risk scores for multiple adverse outcomes (e.g., acute kidney injury, prolonged ventilation, stroke) based on data in the patient’s electronic healthcare record, including demographics, patient risk factors, laboratory results, vital signs, clinical observations, and medications. Our field study is the first step in a long-term multidisciplinary project aiming to develop, deploy, and evaluate a predictive AI system in the CT-ICU.

Our work emphasizes user-centered design and understanding of stakeholder needs. These practices are considered crucial to the design and successful adoption of AI systems in healthcare (16–20). Further, our work treats AI as one component within a sociotechnical system, as advocated by other researchers (21, 22), and investigates sociotechnical factors likely to influence AI adoption in the CT-ICU.

While many predictive analytics are being developed for healthcare, few have been assessed in clinical trials; most focus on algorithm development using historical data rather than live deployment in practice, involve just a single predicted outcome, and are not in the CT-ICU setting. Moreover, there has been very limited investigation into human-interface and workflow needs related to AI in CT-ICUs. Thus, a significant gap remains between AI algorithm research and successful deployment and adoption in CT-ICU settings. To design effectively for this context, we first need to gain a deep understanding of current practices, clinical team needs, factors influencing AI value and trust, and potential barriers to adoption. Our field study addresses these gaps through the following contributions: (1) we characterize the roles, work practices, challenges, and technology opportunities surrounding patient data monitoring in the CT-ICU; and (2) we characterize tasks, requirements, and potential barriers to adoption for a system that predicts adverse patient events in a critical care setting.

By elucidating practices, challenges, barriers, and opportunities, our research offers insights useful for the design and deployment of AI tools in healthcare. The results of our single site study set the stage for the design and development of algorithms and interfaces, as well as for clinical trials of predictive AI systems in the critical care setting, with potential for broader applicability to technology design in related healthcare environments. We synthesize our findings as a preliminary, empirically grounded, proposed framework (*Trusted AI for Clinical Teams (TACT)*) for development of AI technology in healthcare, with a focus on design for clinical team trust and adoption.

## 2 Related work

Previous research on the development and implementation of predictive analytics in healthcare and intensive care settings focuses on four key areas: user needs and trust evaluation (23–25), predictive analytics in healthcare settings (26–29), technological aspects of development and implementation (30–32), and frameworks for AI technology development and implementation in healthcare settings (33–35). Each area is critical to advance computational medicine. However, there is a current gap in the literature on understanding the unique needs of a healthcare team in the CT-ICU setting. Our study focuses on identifying user needs and barriers to implementing a predictive analytics system in the CT-ICU, identifying features important to the healthcare team, and discussing implications for developers. In addition, while there are several frameworks for the development and implementation in the healthcare setting, there is a lack of AI development frameworks with a focus on clinical team trust and adoption of AI technology. As an outcome of our field study, we develop a suggested framework for AI development to support clinical team trust and adoption throughout the development process.

In work similar to ours, Benda et al. (23) conducted a qualitative study across three healthcare organizations, interviewing stakeholders (n=49) in ambulatory multispecialty practices about the implementation of a predictive analytic decision support. The study sample size was large, with participants including administrators (n=25), health informatics personnel (n=7), and end users (n=15). The end users represented the clinical team, including the following roles: primary care provider (including practice leads), hospitalist, nurse practitioner (primary care), care manager (RN), health coach (RN), and case manager/social worker. Thematic analysis indicated that decision support needed to be clear and actionable, incorporated into current workflows, and emphasized the importance of trust, credibility, and interpretability (23). While there are commonalities, our study focuses on the frontline clinical team as primary end users, to gain clarity and additional insight on the needs and barriers of this population. More importantly, our study focuses on a clinical team in an in-patient, CT-ICU, critical care setting, as opposed to an outpatient ambulatory setting, where the needs of the clinical team may be unique. In addition, we had similar findings regarding the need for clear, actionable predictions that fit current workflows, but our study delves into the unique needs of a CT-ICU and identifies insights for developers in predictive analytics workflows and interface design.

In a literature review to understand user trust in AI-based clinical systems, Tucci et al. (24) identified several key factors that influence trust in AI-based diagnostic and clinical decision support systems among healthcare professionals. These factors include explainability, transparency, interpretability, usability, and educating the end users. Our study expands these findings, by amplifying healthcare professionals’ concerns on the importance of trust for adopting AI systems in the CT-ICU setting and identifying which factors influences the increases or decreases trust in these systems, as key considerations for development and implementation.

Past studies have examined barriers and acceptances of clinical decision support system (CDSS) in clinical contexts. For example, in a mixed-methods study of physicians (n=14) in the Emergency Department by Fujimori et al. (36), the acceptance, barriers, and facilitators to implementing a CDSS for a real-time AI that predicts aortic dissection were investigated. While attitudes toward the system were favorable, several barriers were identified, including concerns about system performance, alert fatigue, compatibility, workflow disruption, evidence strength, and design quality. Similarly, a study of an AI-based radiology system by Zajac et al. (25) described clinical usefulness as a key element informed by the AI functionality for radiologic interpretation, AI decision thresholds for alert, AI medical focus or what is predicted, and AI explainability (25). They recommend allowing users to select AI functionality, radiological functions, and AI threshold adjustments (at the clinic and personal levels). Although such studies directly evaluate AI-based systems, none of them investigate AI in a CT-ICU context.

## 3 Methods

We conducted a field study in the CT-ICU in a large academic medical center. Our approach consisted of semi-structured interviews with key healthcare team members (Table 1) and observation of key events (Table 3).

**Table 1:** Participants.

Participant group	Subgroup	Number	Interview structure
Nursing	Newer nurses	3	
	Experienced nurses	4${}^{*}$	Semi-structured interviews${}^{d}$
Advanced Practice Providers (APPs${}^{b}$)	NP/PAs${}^{a}$	3	Individual interviews${}^{e}$ (10/14)
Physicians	Surgeons	2	Observational interviews${}^{\;f}$ (4/14)
	CVCC${}^{c}$ Attending Physicians	2	

Participants included Nurses, Advanced Practice Providers, and Physicians in a single CT-ICU. Total participants (n=14).

${}^{*}$Including 2 nursing supervisors + 2 experienced bedside nurses.

${}^{a}$Physician Assistants (PA) or Nurse Practitioners (NP); given the small number of participants and similar function in the CT-ICU, PAs and NPs were not differentiated.

${}^{b}$APP: Advanced Practice Provider (APP) is the institutional terminology for Physician Assistants (PA) and Nurse Practitioners (NP).

${}^{c}$CVCC: Cardiovascular Critical Care Attending.

${}^{d}$Interviews were semi-structure; see the Supplementary Material for the interview guide. A short description of the project was provided to all participants as outlined in the Staff Meeting Introduction Guide of the interview guide in the Supplementary Material. No prototypes or vignettes were used.

${}^{e}$Individual interviews were conducted in a private room with only the interview team and participant present (2/3 newer nurse, 1/4 experienced nurse, 3/3 APP, 4/4 physician interviews).

${}^{\;f}$Observational interviews were conducted individually in the CT-ICU while the participant was working (1/3 newer and 3/4 experienced nurse interviews). There was minor overlap with a nursing supervisor during the introduction for observational interviews. During analysis, comments of each respective participant were attributed to that participant.

### 3.1 Research questions

Our overarching research question (RQ) was to understand needs of the healthcare team for implementation of predictive analytics in the CT-ICU. Specific research questions were as follows:

- RQ1: What current practices in the CT-ICU could impact AI use and how?
- RQ2: What are the attitudes toward predictive analytics in the CT-ICU?
- RQ3: What barriers may hinder implementation of predictive analytics in the CT-ICU?
- RQ4: What technological features are important to predictive analytics end users in the CT-ICU?

### 3.2 Positionality statement

As researchers, we are an interdisciplinary team with diverse experiences and perspectives. Carey Barry is a licensed and board-certified Physician Assistant (PA) and PA educator with clinical experience in surgical settings, including cardiac and vascular surgery. Carey is also a PhD student in personal health informatics with a focus on data visualization and human-computer interaction. Sydney Purdue is a PhD student in Interdisciplinary Design and Media with a focus on data visualization, specifically data physicalization and situated visualization; her background is in statistics and art. Matthew Goodwin, PhD, is a behavioral scientist with research and clinical experience working with children and adults on the autism spectrum with a research focus in personal health informatics, computational behavioral assessment, ambulatory psychophysiology, and machine learning. Mahsan Nourani, PhD, Clifton Forlines, PhD, and Melanie Tory, PhD, are computer scientists focusing on human-computer interaction and human-centered AI.

### 3.3 Participants

CT-ICU care teams typically consist of cardiothoracic surgeons, critical care physicians, medical residents and fellows, Physician Assistants/Associates (PA), Nurse Practitioners (NP), nurses, and clinical pharmacists (1). Members of the care team in the academic medical center CT-ICU, where interviews were conducted, reflect the typical CT-ICU care team. Through discussions with CT-ICU leadership, key interviewees were identified, including surgeons, cardiovascular critical care (CVCC) attendings, advanced practice providers (APPs), including PAs and NPs, nursing supervisors, and bedside/staff nurses (Table 1).

Initial participant recruitment occurred through two contacts in the CT-ICU. The research team attended a virtual staff meeting before the study to introduce the project and answer questions. Once the interview team arrived in the CT-ICU, staff were invited to participate and informed that participation was optional. Each participant verbally granted informed consent. Participant selection was a combination of purposive (identified role categories to include) and convenience (specific individuals on shift during the field visit). Fifteen people were invited to participate, one declined. No participants dropped out and no interviews were repeated. The research team had no prior relationship with the participants outside of this study.

Participants were representative of CT-ICU team members with varied roles, ages, genders, levels of critical care experience, and comfort with technology. Fourteen (n=14) participants were interviewed, including nurses (n=7), advanced practice providers (APPs) (n=3), and physicians (n=4) (see participant Tables 1, 2). Given the small sample size, age and sex were not included for each participant to protect privacy. Participants’ mean age was 37.8 (range 26–53). Participants self-identified sex as female (n=6) or male (n=7), and one participant did not disclose their sex. Participants’ critical care/ICU healthcare experience ranged from 1 month to 22 years (mean=4.6 years).

**Table 2:** Participant background.

No.	Category	Role	ICU experience classification	Comfort with technology
P1	Nurse	Clinical Nursing Coordinator	Experienced	Moderate to high comfort level. “Middle of the road” in comfort with technology.
P2	Nurse	Bedside Nurse	Experienced	Lower comfort level. “Takes a while to learn something new.”
P3	Nurse	Bedside Nurse	Experienced	Lower to moderate comfort level. “Depends on the technology.”
P4	Nurse	Bedside Nurse	Newer	Moderate comfort level. “Would rate myself a medium.”
P5	Nurse	Bedside Nurse	Newer	Lower comfort level. “Not super tech savvy.”
P6	Physician	CVCC${}^{c}$ Attending	Experienced	Moderate to high comfort. “Comfortable, but not an expert.”
	PA or NP${}^{a}$	APP${}^{b}$	Experienced	High comfort level. “Very high comfort level with technology, everyone comes to me with questions”.
P8	PA or NP${}^{a}$	APP${}^{b}$	Newer	Moderate to high comfort level. “Pretty comfortable.”
P9	PA or NP${}^{a}$	APP${}^{b}$	Newer	High comfort level. “Pretty comfortable”, “I like gadgets”, “comfortable with devices and systems.”
P10	Nurse	Bedside Nurse	Newer	Moderate to high comfort. “Pretty comfortable”, “I learn relatively quickly”.
P11	Physician	CVCC${}^{c}$ Attending	Experienced	High comfort level. “Pretty comfortable” and “tech savvy.”
P12	Physician	Surgeon	Experienced	Comfort level not obtained.
P13	Physician	Surgeon	Experienced	High comfort level. “Very comfortable with technology.”
P14	Nurse	Clinical Nursing Coordinator	Experienced	High comfort level. “Fairly comfortable” with technology.

Participant background and technology comfort level.

Participant identifying information was removed and participants were identified by participant number (No.). Information collected included training category (Category), current role (Role), critical care experience (ICU Experience Classification), self-described comfort level and technology use. Team members with with over 3 years of experience were classified as experienced based on participant judgment and prior nursing literature (37).

${}^{a}$Physician Assistants (PA) or Nurse Practitioners (NP); given the small number of participants and similar function in the CT-ICU, PAs and NPs were not differentiated.

${}^{b}$APP: Advanced Practice Provider (APP) is the institutional terminology for Physician Assistants (PA) and Nurse Practitioners (NP).

${}^{c}$CVCC: Cardiovascular Critical Care Attending.

Participant critical care experience level was classified as *newer* (<3 years of experience; n=5) or *experienced* (3+ years of experience; n=9). Experience classification was based on participant judgment and prior nursing literature (37). Participants’ self-reported comfort with technology included low (n=3), moderate (n=5) and high (n=5) groups. See Table 2 for participant details.

### 3.4 Observations and interviews

Before the interview sessions, members of the research team attended a virtual staff meeting to introduce the project and answer any questions. A short description of the project was provided to all participants during the staff meeting as outlined in the *Staff Meeting Introduction Guide* section of the interview guide in the Supplementary Material. Data collection was conducted over three consecutive days in November 2023, totaling 25 h of interviewing and observation time in the CT-ICU. The study schedule included key events and activities outlined in Table 3. Two researchers jointly conducted all interviews and observations, [C.B.] and [S.P.]. Individual interviews were conducted in a private space adjacent to the CT-ICU or in the CT-ICU itself. Semi-structured interviews were conducted using an interview guide (see Supplementary Material) to structure the conversations. No prototypes or vignettes were used. Key events and activities in the CT-ICU were identified and observed, including patient transfer to the CT-ICU from the operating room (OR) after surgery (described by the team as “landing a heart”), interdisciplinary rounds, shift change, day shift, night shift, and staff interactions/reactions to alarms and alerts (Table 3). Audio recording was not allowed in the CT-ICU to preserve patient privacy but was allowed in the private interview space. We obtained verbal permission from each participant to audio-record their interview, as applicable. Photos and video recordings were prohibited. Written notes and sketches were recorded by the interview team during event observations in the CT-ICU, observational interviews conducted in the CT-ICU, or if a participant declined audio recording (5/14 interviews). Written notes were recorded by both onsite interview team members during the interviews and observations.

**Table 3:** Observed events.

Observed events	Number observed
Staff meeting—virtual	1 (Introductions)
Interdisciplinary rounds	1
Landing a heart${}^{a}$	1
Reaction to an alarm	Numerous
Change of shift	1
Day shift	3
Night shift	1

ICU events and activities observed during the study.

Based on preliminary discussions with the CT-ICU, specific events were identified as key points in communication exchange in the CT-ICU. These events were intentionally observed.

${}^{a}$Landing a heart is a term used by the CT-ICU team to describe the process of transferring and admitting a patient to the CT-ICU from the operating room (OR) after surgery.

### 3.5 Data analysis

Written notes from the on-site team were consolidated and typed into Microsoft Word for the observed events (Table 3) and non-audio-recorded interviews (5/14 interviews). Notes were not recorded at the Virtual Staff Meeting, as the purpose of the session was to introduce participants to the study. Audio recordings were transcribed using the transcription feature in Microsoft Word and manually edited for accuracy (7/14 interviews). All transcriptions and notes were imported, and qualitative data analysis was conducted using NVivo 14.

Coding and thematic analysis of interviews was conducted using the approach described by Braun and Clarke (38, 39). A single coder with clinical domain knowledge conducted the primary coding and thematic analysis in alignment with the reflexive thematic analysis principles described by Braun and Clarke (39). The coder read through all written notes, transcribed notes, and listened to all audio recordings before coding. The codebook and examples were reviewed with a senior author with human-computer and human-centered AI expertise to ensure depth and nuance of analysis, manage the coder’s domain knowledge, and ensure interdisciplinary expertise was leveraged. We conducted iterative review at regularly scheduled biweekly meetings, where we discussed the evolving codebook and examples and came to consensus on coding of unclear cases. Observational data were not explicitly coded but were used as context for interpretation of the interviews. The coded data were evaluated across categories including current practices, sentiments, barriers, and desired features to generate themes and key insights.

Coding was refined through an iterative process, with a focus on generating themes in current practices in the CT-ICU setting; team needs and barriers to implementing predictive analytics in the CT-ICU setting; important design features for predictive analytics and user interfaces; and assessing attitudes toward AI in the CT-ICU setting. Each transcript was coded multiple times, with the initial pass involving open coding and subsequent reviews involving selective coding. Subsequently, deductive framework mapping (40) was used to map the findings to the Technology Acceptance Model (TAM) constructs of *perceived ease of use* and *perceived usefulness* (41). TAM was selected due to its wide acceptance and comparability. A Consolidated Criteria for Reporting Qualitative Research (COREQ) Checklist (42) was completed, and it is provided in the Supplementary Material to support transparent reporting.

## 4 Findings

The CT-ICU is a challenging place to work, given high-stakes outcomes, time pressure, and a cacophony of data and alarms. Introducing new tools into this environment is exceptionally challenging, particularly AI tools that are often poorly understood black boxes surrounded by uncertainty. Our results generated several key themes relevant to the implementation of AI tools, including key thematic findings discussed in detail in the following sections. For a summary of key findings, see Figures 1, 2.

**Figure 1:**
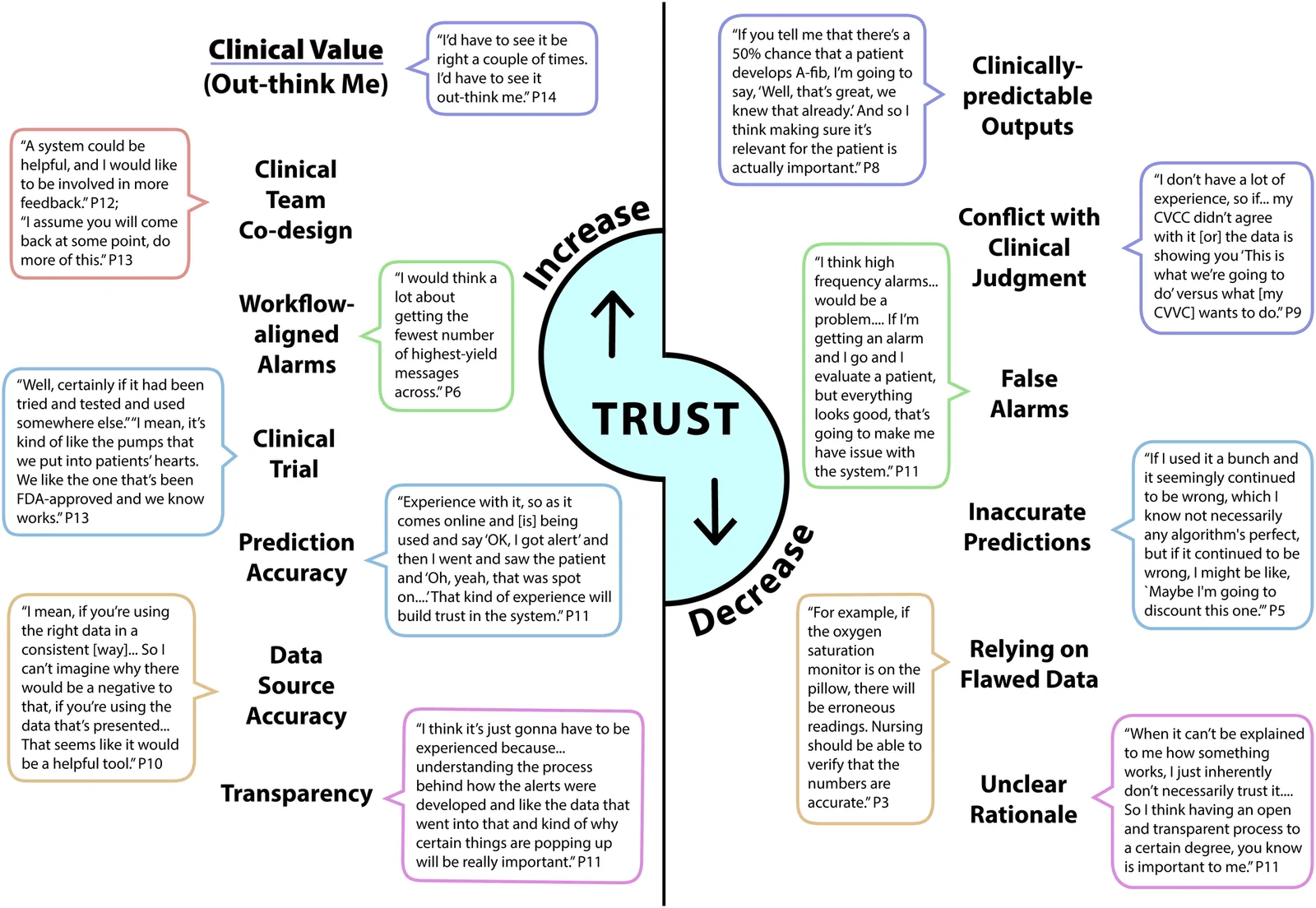
Identified factors that would likely increase or decrease clinical team trust in AI in the CT-ICU, paired with a representative participant quote. Factors identified to increase trust included: added clinical value; engagement in clinical team co-design; workflow-aligned alarms and alerts; prediction accuracy or proven accuracy in clinical trials; data validation and accurate data sources; and transparency in predictions, including the availability of top ML features contributing to the prediction. Many of the factors reducing trust in an AI system were opposed to those increasing it. Factors identified to decrease trust included: outcomes that are clinically predictable given current clinical judgment; predictions that contradict current clinical judgment; frequent false alarms; inaccurate predictions; reliance on the system or training on flawed data; and predictions with no clear rationale. Among all factors, *added clinical value* was the most important in influencing trust in AI.

**Figure 2:**
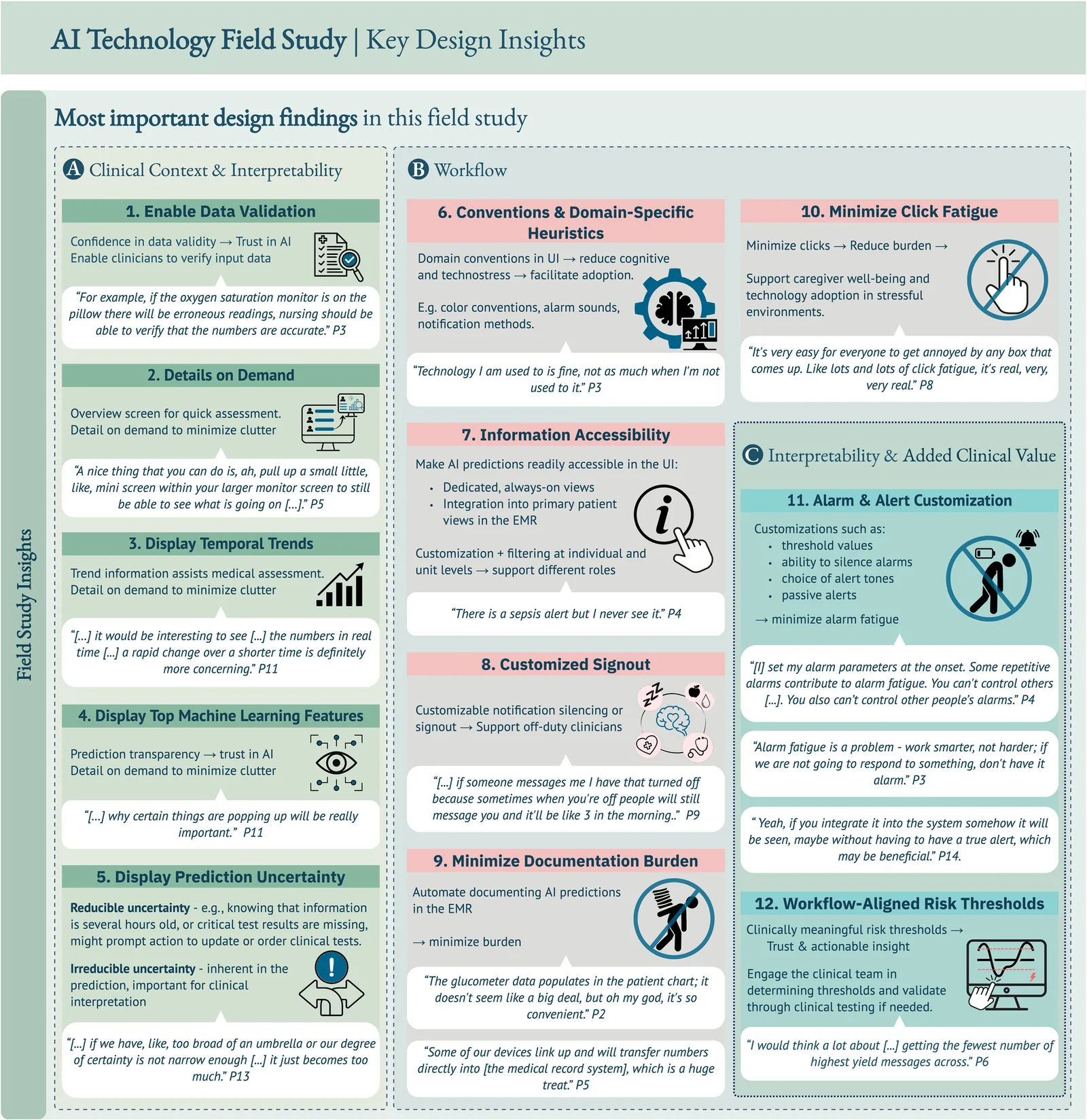
Key design insights and sample quotes show the most important findings for design of AI technology in the CT-ICU setting. Key design insights fell under broader themes of design elements that influence **(A)** clinical context and interpretability, **(B)** workflow, and **(C)** workflow factors affecting added clinical value. Factors influencing clinical interpretability in panel **(A)** include (1) enabling data validation, (2) showing details on demand including (3) temporal trends and (4) top machine learning features contributing to a prediction, and (5) showing prediction uncertainty, including reducible and irreducible uncertainty. Factors influencing workflow in panel **(B)** include (6) using conventions and domain-specific heuristics, (7) ensuring information is accessible in the context of the workflow, (8) considering customized sign-out features, (9) integrating into electronic medical records to minimize documentation burden, and (10) minimizing click fatigue. Nested in workflow are factors in panel **(C)** that can impact the perceived added clinical value of the AI technology: (11) alarm/alert customization and (12) work-flow aligned risk thresholds.

One of the most significant aspects of AI in the CT-ICU among all members of the clinical team was the concept that any AI technology in the space must have *added clinical value* to the clinical team and patients, while minimizing additional *workflow burdens*. As we discuss in our findings, workflow burdens are not only a challenge to the team; they can have real safety implications for patient care.

In addition, we explore the needs and barriers of the clinical team for the implementation of AI in the CT-ICU. Providing means for the clinical team to establish trust in the AI system, such as through co-design processes, transparency in the development and predictions of an AI system, and consistent prediction accuracy were meaningful to the clinical team. We identified steps that can be taken to improve integration into workflow to help reduce additional burdens, including robust onboarding, establishing workflow-aligned alerts and alarms, establishing workflow-aligned risk thresholds, integrating charting to reduce charting burden, and developing user interfaces with conventional user interface heuristics and reducing click burden. We identify technostress as an additional consideration when introducing an AI system in the CT-ICU and strategies to reduce technostress and promote personal wellness in this space.

### 4.1 Attitudes towards AI use in the CT-ICU

Care team attitudes towards AI (RQ2) are likely to influence technology acceptance. Among our participants, the general attitude toward AI and predictive algorithms could be described as cautiously optimistic, with thoughtful skepticism. Participants expressed excitement about the potential uses of AI in the CT-ICU and considered it a useful technology.

“It is so important these people are making strides every day. If we are not making progress, we are falling behind.” P3.

“As a new nurse, I think… I would definitely benefit from something like that.” P10

“I think it’s a great idea. I think we just have to feed it the right data…. I think it can be a very powerful tool. I think there’s going to take some time to sort of dial it in, like, what system works best for everybody.” P14

The clinical team also expressed that they were willing and interested to participate in the process of developing a tool that would be most useful to the clinical team in providing patient care. There was also a sense of value in clinician involvement and feedback throughout the project’s progression.

“It’s an exciting concept. Like I said, I’ve wondered how to make this more efficient, more successful, and continue to reduce error.” P7

“A system could be helpful, and I would like to be involved in more feedback.” P12.

While there was much excitement about the potential of an AI tool for patient care, several concerns were raised about the system, primarily regarding potential negative impacts on workflow, its accuracy, and a potential lack of *added clinical value*. Participants saw added clinical value as particularly critical because a system that is perfectly accurate at telling the care team what they already know just adds unnecessary effort.

“This has the potential to create more noise without helping.” P12

“…sometimes you guys will do the numbers and you’ll be like the accuracy of this is unbeatable …we’ll trust you for a little while, and then, you know, after a couple of months, we’ll determine for ourselves, like, do we clinically experience it to be that accurate or not.” P6

These results suggest that careful attention to the clinical team’s needs, concerns, and potential barriers to implementation, through an iterative design process with clinical team feedback, is important for building trust and understanding of AI systems. We expect these activities to promote a bridge from research to clinical implementation. Additional aspects of *added clinical value* are discussed in Section 4.2.1.

### 4.2 Clinical team AI use needs and barriers

Our results strongly emphasized the need for added value, trust in AI, and additional workflow burden as key barriers to overcome for AI adoption. The concepts of added clinical value and CT-ICU workflow are complex and multifaceted. Deeper insights into factors contributing to each concept are identified and discussed in detail in the subsections below and summarized in Figures 1, 2. This theme addresses RQ1 (current practices), RQ3 (barriers), and RQ4 (important AI tool features).

#### 4.2.1 Added clinical value

One of the most important concepts for participants was *added clinical value* (see also Figure 1 (left), Figure 2C). Given the high acuity of patients and the demands on the clinical team, any new tool introduced into the CT-ICU must add value to patient care or clinical functions. Participants consistently expressed that an AI system introduced in the CT-ICU setting must be highly accurate and provide knowledge or insights beyond what is already known to the clinical team.

“I’d have to see it be right a couple of times. I’d have to see it out-think me.” P14

“If we clinically find it super useful, then every time I get an alert, I’m going to log right into a computer. If I’m getting reds [critical alerts] once a week and they turn out to be reliable, and that’s of huge value.” P6

The concept of added clinical value is complicated, can be greatly influenced by the design of the user interface for AI predictions, and encompasses a variety of considerations, including determining the best thresholds for risk and thus alerts, and the impact of false positives or false alarms on the system’s clinical value.

“Noise is an issue; the challenge is to distill it into a quiet and important signal.” P12

Participants indicated that the alert threshold should be high, but no agreement on an exact number. The confidence percentage range for a useful threshold was from 75% to 100%. This speaks to the risk and impact of introducing increased alert and alarm fatigue in the setting.

“The confidence has to be like 99.999999%, otherwise I’m going to ignore that 100% of the time.” P6

“I think it probably depends on the situation, but I think a confidence above 75 would be strong.” P8

“Like 89%, 85%, 90%…. I don’t know if I’d really care to know if it was like, 50% chance of this happening or even 60% still doesn’t seem like… there’s just so much going on that having another thing added to it, if it’s not really positive… or pretty strong ‘going to happen,’ that I feel like would be too much.” P10

In addition to determining the appropriate risk and alert thresholds, conveying uncertainty was expressed as an important consideration in the design, influencing how the predictions are interpreted and perceived.

“If we have, like, too broad of an umbrella or our degree of certainty is not narrow enough… it just becomes too much.” P13

“[I] would want sensitivity and specificity. Confidence interval[s] would be helpful.” P12

The timing of predictions and alerts also contributes to *added clinical value*. Participants discussed current AI tools used in clinical settings, specifically those for sepsis prediction. Although an AI sepsis prediction tool was available, participants were not using it. The lack of added clinical value in this tool was multifactorial, related to the validity of the data (discussed in 4.2.2.1), the tool’s accessibility within the workflow (discussed in 4.2.3.2), and the timeliness of the notifications.

“I’ve worked places that have had, like, the normal sepsis thing, where it’s like you see the change in vital signs or these labs, but… its like 4 h later. So, it’s data that’s old, or it’s data that might not be pertinent now, so… the patients could be better now, or they could have already… gone through this event.” P9

The need for actionable insights is another contributor to *added clinical value*. When ordering diagnostic studies in the clinical setting, clinicians are trained to consider: *Will this change the care we give to the patient?* or *What am I going to do with these results?*, before ordering a test. AI algorithm predictions can be considered in the same context as a diagnostic study. The likelihood of actionable insights influences the clinical value without becoming *noise*.

“I would think a lot about, like you know, getting the fewest number of highest yield messages across.” P6

For real-time predictions, trends over time is a helpful metric.

“I would want to see. Well, it would be interesting to see, kind of, the numbers in real time… if I got an alert and then I maybe would go back later and follow up to see, oh, that person’s score has come down. […] maybe they’re getting better, but… a rapid change over a shorter time is definitely more concerning.” P11

The results above strongly suggest that careful consideration is needed for evaluating accuracy, establishing workflow-aligned thresholds for risk and alerts, determining alert timing, and developing a user interface that easily conveys prediction certainty and uncertainty, as well as trends over time. Each of these factors will influence the perception of the *added clinical value*. In addition, the current practices for using risk categories in the clinical setting must be considered. Table 4 outlines three categorical levels described by our participants to quantify patient risk, with associated color coding: critical (red), moderate (yellow), and low (blue). Alignment with current practices may improve integration with workflows and perceived ease of use, thereby enhancing perceived *added clinical value*.

**Table 4:** Hierarchy of alerts.

Risk to patient	Nomenclature	Auditory alarm	Visual alarm	Communication
Critical	Red	Loud and rapid	Red visual on the Phillips patient monitor	Direct conversation or page to provider.
Moderate	Yellow	Moderate; lower and less rapid than red alarm	Yellow visual on the Phillips patient monitor.	Mixed, depends on urgency. Epic chat vs. call/page.
Low	Blue	Quieter and slower tone	Blue visual on the Phillips patient monitor.	Often none. Epic chat feature.

The CT-ICU that we observed had an unofficial hierarchy of alerts and alarms related to the danger to the patient. Participants referred to these levels as“red”, “yellow”, and “blue”.

#### 4.2.2 Trust in AI systems

Several key factors that could affect trust in the AI system were identified by our participants. See Figure 1 for a summary of themes that impact clinical team trust in AI with example quotes.

Participants indicated that the system’s ability to accurately predict events with minimal false positives or false alarms would increase their trust in it. In addition to empirical evidence of prediction accuracy, evidence of accuracy through clinical testing or clinical trials would increase trust in an AI system.

Providing opportunities for clinical team feedback and involvement during system development and the iterative design process, with transparency about how and why the system makes a prediction, would improve trust in the system and allow clinicians to make their own judgment about the system’s accuracy in a given situation.

Participants valued knowing why the predictions were made as a source of trust in the system and as a way to add clinical value.

“This patient, you know, has not [received] adequate nutrition for a week now, they’re not moving as much, these lab levels are… you know, and so it looks like there might be an increased risk for a pressure injury” P5

Our findings suggest that showing the top reasons for any prediction allows the clinical team to evaluate the validity of the source data and inform their clinical assessment. Including the top machine learning features as detail-on-demand in the user interface would allow the clinical team to access this data without cluttering the primary view.

##### 4.2.2.1 Data validity and erroneous data

The potential for erroneous data to influence AI recommendations was seen as a critical concern; staff expressed that they would need confidence in the validity of the underlying data as a prerequisite for building trust in a predictive AI system (see Figure 1 (right), Figure 2A). Data validity was important at both the individual patient level and the AI system training level. Both technological and human errors were identified as concerns in patient data collection and documentation. The CT-ICU team is trained to detect technological errors and investigate data in the context of the overall clinical situation, including patient examination. Participants expressed that data validation becomes important in this setting and is a standard part of clinical practice.

“For example, if someone is brushing their teeth or burping a baby and the rhythm monitor alarms VT [Ventricular Tachycardia], this is [an] artifact.” P3

“My fear, of course, is on the other side. Just like I have very powerful tools at my disposal, you know, they’re not always valid for every patient in every scenario, so you’re still going to have outliers…. How do you tell the machine that the data it’s getting may not be valid? And then how would it adapt its predictions based on that? Because I can look at that and I can validate those things and I can look at other things to validate my findings, but I don’t know if the machine… Is there enough to have that thought process as of yet or how that would change things, right?” P14

Human error was also a concern regarding patient data. There may be a delay in logging patient data in the medical record or data-tracking systems.

“If it is very busy, nursing can get behind in charting the results.” P1

In addition to delayed data logging, the cadence of checking patient vitals or recording data is not minute-to-minute and may occur only every few hours.

“But then you’re also relying on this data, where the nurses only get the vitals every… I don’t even know what frequency it is, maybe every few hours. So the lag time is… I don’t really know if we’re talking about the most important things if there really is a lag time.” P13

This lag in patient data may leave an AI system without the most up-to-date information, affecting real-time data analytics and leading to inaccurate or delayed predictions, reducing clinical value. This suggests that data freshness and the presence of missing or out-of-date data values may be important components of any predictive AI explanation. Further, an AI system may not know that the data contains errors or that the clinical team is experiencing delays in logging. A data validation process may need to be built into the system and the onboarding process to ensure predictions are made with accurate data. Warning messages and uncertainty information may be needed to indicate when system predictions are missing data or based on data older than some set threshold.

“[We] shouldn’t be notified… until it is validated.” P12

The order of notification and data validation is also important. Nursing participants often validate data before physicians and APPs are notified of a clinical issue, to minimize false alarms that reach all team members, especially for data known to the clinical team to be prone to false alarms. Creating customizable data validation checks in the interface may help reduce false alarms caused by inaccurate data.

In addition to concerns about individual patient data, participants recognized the importance of training data set validity for machine learning. Not only are there known logging errors, but there are also unique considerations for the patient population that may affect how a machine learning algorithm performs. Participants had concerns about specific symptom profiles that might “confuse” an AI algorithm.

“Typically you can tell prior to the machine; [the algorithm] can confuse it with cardiogenic shock.” P4

“I think the algorithm is skewed in our population… sepsis can look very similar to cardiogenic shock, which is also really commonly seen in our population, so I think that might also skew the algorithm.” P14

In the CT-ICU, patients face unique risks associated with their underlying medical conditions. At the time of our interviews, a sepsis predictive analytics tool was available in the clinical setting through the electronic medical record but participants reported it is rarely used in the CT-ICU. Participants reported that the predictions did not outperform their own clinical acumen and the perceived accuracy was low. This sentiment aligns with recent literature showing low added value in a widely used sepsis prediction model (43). Participants reported the sepsis algorithm was the same as that used across the inpatient setting, and participants felt that the lack of accuracy in their patients was likely due to the unique conditions of the CT-ICU population. Tailoring training datasets to local or comparable populations and involvement of healthcare providers in the iterative design process may help address this issue.

##### 4.2.2.2 Role of AI: actionable support for clinical judgment

Participants emphasized the importance of clinician-led decision-making in clinical settings (summarized in Figure 1 (right)). P12 expressed that AI in healthcare should be “adjacent to the art of human clinical medicine” rather than supplemental. All initial, and arguably all, implementations of AI systems and AI predictions in the clinical setting will require evaluation and interpretation by the clinician with a human-in-the-loop model.

“I wouldn’t trust it more than my clinical judgment.” P4

Further, participants wanted clarity on the purpose of the AI system. At a high level, participants wanted it to be clear which role the AI will support in the clinical setting.

“What am I trusting it for?” P10

This highlights the importance of clearly defining the system’s purpose and discussing actionable insights from the results when developing and implementing an AI system in clinical settings. Our participants consistently expressed that a predictive AI system should be designed as diagnostic support, aimed at augmenting clinical judgment rather than replacing it.

When discussing trust in an AI system, participants wanted clarity on the system’s purpose and whether predictions would yield actionable insights or be associated with a *clinical guidelines* (defined steps to take based on the prediction). In addition to clearly defining the role of diagnostic support predictions, it may be crucial to define associated clinical management guidelines.

“What do I want to do about it? …Do you want me to draw some labs? Do you want to give him more blood pressure [medications]? What should I do about this thing, you know?” P14

“It needs to be relevant and acted upon.” P12

In clinical practice, the healthcare team continuously evaluates a variety of patient-related signals and makes decisions based on clinical judgment, evidence-based medicine, and patient-centered practices; the interventions and actions taken by the team are grounded in the same principles. In some cases, evidence-based clinical guidelines or nursing protocols are developed to guide the clinical team’s actions in response to specific signals or combinations of signals. A signal that does not require action can become *noise*.

In some ways, AI predictive models can be considered like other patient testing in the medical setting. For example, lab or radiographic tests are run and interpreted by the clinical team. Decision-making is then executed by the clinical team in the context of evidence-based medicine practices and for each individual patient. Similarly, the clinical team will need to evaluate AI predictions and the factors that contribute to the predictions.

As with all evidence-based medical practices, the development of clinical guidelines or nursing protocols to accompany machine learning predictions would benefit from clinical trials demonstrating improved outcomes over the current standard of care. This requires an iterative design and implementation plan. To avoid becoming *noise*, clinical trials must demonstrate added clinical value to patients and care teams through improved outcomes or workflow.

#### 4.2.3 Workflow burden

The workflow in a busy critical care environment is complex and high-stakes. Many workflow-related factors were identified as areas of concern with the introduction of AI in the critical care setting; examples and actions to address these burdens are summarized in Figure 2B. The theme of workflow burden was consistent among all participants 14/14), with alarm burden, fatigue, task burden, technology burden, and personal burden and wellness as thematic concerns.

##### 4.2.3.1 Alarm burden and Fatigue

Alarm fatigue was by far the most prominent theme across all participants and a potentially significant barrier to AI adoption (RQ3). All (14/14) participants spoke about alarm fatigue, most by name and others by description.

“About 85% of the alarms are false, but you need to check a lot of false alarms. You are obligated by ethics to check every one of them.” P4

[When all the alarms are going off] “…it’s like I can’t think! You know how when you are driving and you turn down the radio to [concentrate], you can’t turn them off.” P4

“This has been a big annoyance on the unit. I don’t know if it’s an issue with the monitor that can be fixed… but we’ve had issues where the monitor is reading the patient’s rhythm. We’re looking at it elsewhere and the rhythm’s fine, but it’s saying something totally different. That’s not accurate and it’s just the machine. It’s not the patient.” P5

“The telemetry alarm… most providers have alarm fatigue from that because… the machine is not very good at differentiating rhythms.” P6

“It will keep alarming after a couple of minutes, which can be annoying on the floor if it keeps reading/misreading something.” P8.

“There’s this thing called alarm fatigue, where they’re in your face all the time, right? We lose track of them.” P14

Providers were consistently concerned that AI algorithms could contribute to alarm fatigue by providing yet another metric that would generate alarms. They reported that an alarm generated by the AI would only be useful in the event of a high-risk event, suggesting that careful threshold setting would be needed to ensure that predictive AI systems do not further contribute to an already significant burden. Alarm customization and the silence feature were valued for helping reduce alarm fatigue. Participants 4 and 8 explained that some critical alarms cannot be silenced for more than five seconds.

“Alarm fatigue is a problem. Work smarter, not harder; if we are not going to respond to something, don’t have it alarm.” P3

“[I] set my alarm parameters at the onset. Some repetitive alarms contribute to alarm fatigue. You can’t control others. Apnea alarms, many patients have sleep apnea, the alarm has low threshold. You also can’t control other people’s alarms.” P4

“So, there’s some patients like that you can’t turn off if it’s a hard stop. So if it reads V-Fib you can’t turn that off and it will keep alarming.” P8

Addressing RQ4 (technological features), these responses suggest that caregivers want control over the customization of alarms generated by an AI system, or perhaps that the AI system should not set off alarms at all, but rather serve as an information system accessible on demand. Useful customizations might include threshold values, the ability to silence alarms for a time period, and the choice of audible signals. The ability to differentiate each alarm in the clinical setting was a skill developed across the clinical team, with a learned response for each tone.

“You become familiar with the different tones of the alarms and quickly learn to locate them.” P1

“If minimum MAP goes less than 60, the alarm is louder and faster and tone is different. There is a clear auditory difference. ECMO and LVAD are different… ‘Boop boop boop…”’ P4

“Where you’ll have like a ‘TING TING TING TING TING’ for a red whereas with a blue alarm, you’ll get like: ‘bep ….. bep …. bep.”’ P5

Participants described a hierarchy of alarms based on the patient’s risk. All team members reported that they could recognize auditory alarms by their sound. The tones are all unique and vary based on the nature of the alert. More serious or critical alarms for conditions that require immediate medical attention are louder, more rapid, and sometimes cannot be silenced. In designing alerts and alarms for AI algorithms in the CT-ICU, careful evaluation of current alerts is important to creating a distinct alert for more critical events. In addition, relying on domain experts to provide the criticality of the underlying condition that triggers the alarm will help determine the appropriate tone, frequency, and other features. There were also preferences for communication methods that varied with the level of alarm (Table 4). Interestingly, members of the care team referred to alarms by color, reflecting the color scheme used for events on patient monitors; any new system may need to conform to the same color conventions for ease of use. Ease of accessibility to data and communication tools during a true alarm should be considered in the software development process.

“So, we get two types of alarms: red or yellow alarms. Red is if the patient’s in like a lethal arrhythmia or an arrhythmia (that’s a change that’s not considered normal) outside of form of sinus. If they all of a sudden go into A-fib or something… it will ring louder with a red alarm.” P5

“The vent alarm sometimes, the tubing is disconnected. The nurse can just plug the tubing back in, and so it doesn’t need to rise to the attending level.” P6

“There are things like some of the blue alarms are things, like, oh, patient’s lead is off, or something seems like it got disconnected, that kind of thing. They wouldn’t even contact me for [that].” P7

These results suggest that AI-driven alarms may need to be developed within a hierarchy of alert criticality levels. Furthermore, for an AI tool intended to be predictive (up to several hours in advance) rather than reactive and urgent (signaling an imminent life-threatening concern), conveying alerts via visual communication or other means, rather than audible cues, may be more appropriate.

##### 4.2.3.2 Task burden: documentation burden and click fatigue

Clinical team members discussed the burden of tasks that take them away from direct patient care and impede their workflow. *Documentation burden* and *click fatigue* are challenging in the CT-ICU and across healthcare settings, representing another potential barrier to the adoption of AI tools (RQ3). Automatic integration into the electronic medical record was valued by the clinical team and was effective at reducing charting burden.

“These are not integrated into [the medical record], and they need to be manually entered every hour. If it is very busy, nursing can get behind in charting…. Anything integrated into [the medical record] is a huge time saver.” P1

“The glucometer data populates in the patient chart; it doesn’t seem like a big deal, but oh my god, it’s so convenient.” P2

“Some of our devices link up and will transfer numbers directly into [the medical record system], which is a huge treat.” P5

Automated alerts are received in the electronic medical record for a variety of patient-related concerns and need to be acknowledged or acted upon by the clinical team. Participants reported a high volume of alerts requiring a “click” to acknowledge or take action. It often blocks them from progressing in their work until it is acknowledged. The alerts can be burdensome and were often viewed as unhelpful.

“We get alerts that way through Epic, but I’d say 99% or more of them are unhelpful and ignored by most providers.” P6

“It’s very easy for everyone to get annoyed by any box that comes up. Like lots and lots of click fatigue, it’s real, very, very real.” P8

“I guess the bigger overarching concern would be making sure that there’s not so many messages that it becomes overwhelming and then we just have message fatigue and burnout, because that happens for sure with the electronic medical record. I mean, I get all these different results for all these different patients, and at the end of the day, I’m just checking them because I have to…. I could kind of see that happening with this.” P13

Concerns about increased documentation burden and click fatigue with the introduction of the AI algorithm were prevalent. In addition, providers were concerned that AI predictions could be buried deep within medical systems and not readily available. Addressing RQ4, this suggests that a dedicated, readily accessible screen, or the integration of the information into primary patient views in the electronic record system, would be important for ensuring that AI predictions are seen and considered in practice without requiring excessive acknowledgments. Participants also worried that they would need to manually document prediction outputs in the patient chart. This suggests value in directly integrating predictive algorithms into electronic medical systems so that AI predictions and evolving predicted risk scores can be automatically documented into the patient record.

##### 4.2.3.3 Technology burden and systems fatigue

With advancements in healthcare technology and health informatics, healthcare workers are experiencing an increased workload burden due to the need to learn and use an increasing volume of technology while providing patient care. Current practice not only requires the healthcare team to develop knowledge of the system, but also additional monitoring, interpretation, documentation, and troubleshooting for each system. Increasing technology can be a burden to the clinical team and a potential adoption barrier (RQ3).

The complex nature of CT-ICU patients necessitates significant use of technology. During an observation with P2 in a patient room, we noted there were six IV pumps, multiple monitors, a continuous venovenous hemofiltration (CVVH) machine, ventilator, Bair Hugger system, computer, bed alarm, and sequential compression devices.

“Well, I interact with the electronic medical record. I interact with the devices that I implant: LVADS, durable and temporary and mechanical circulatory support devices, ECMO, the heart lung machine. I mean, there’s a lot of devices that we’re interacting with, [like] defibrillators, pacemakers. I think it’s kind of a fairly technology heavy sport, if you will. So yeah, I interact with my iPhone continuously, perhaps too much, yeah.” P13

In addition to complicated and numerous patient care technologies, such as left ventricular assist devices (LVAD), extracorporeal membrane oxygenation (ECMO) machinery, ventilators, dialysis machines, pacemakers, and intra- aortic balloon pumps (IABP), the CT-ICU team uses many communication and data-tracking tools (Table 5).

**Table 5:** Communication and data tracking.

Communication tools	Patient data tracking tools
Direct conversation	EMR Chart
Email—MS outlook	PACS Radiology System
Electronic medical record (EMR)/patient chart	Paper Notes
EMR chat feature	SoftID (specimen collection ID system)
MS teams	Window Writings
MS teams chat feature	
Overhead announcements	
Pager	
Phone	
Smartwatch	

Communication and patient data tracking tools currently used by the CT-ICU team.

Perceived comfort with technology may also affect technostress. Participants with lower levels of technology comfort may experience greater technostress when learning new technologies.

“It takes a while to learn something new.” P2

“Technology I am used to is fine, not as much when I’m not used to it.” P3

Our findings show that a rigorous onboarding plan for new technologies in the CT-ICU space is critical to reducing technostress and to using the tool effectively. Consideration of workflow, technology, and current practices in the critical care setting is essential when developing a tool and user interface. Following conventional heuristics to reduce the cognitive load of learning a new technology may reduce technostress and improve adoption.

##### 4.2.3.4 Personal burden and wellness

Several participants expressed the burden they experience when they are too connected to work-related devices. They spoke about the importance of being present while caring for patients and in their personal time. This section addresses RQ2 (attitudes) and RQ4 (technological features).

“I think [smartwatches] are annoying. I mean, first of all, I don’t want to wear it. Second of all, I’m already tied to technology enough and I just… technology is great, but also a lot of it takes you out of being present, you know? And so, for me the watch just takes you out of being present.” P6

Some participants discussed the importance of a short lunch break, during which they could sign out to another colleague and take a mental break.

“So, as a new grad, all the experienced nurses tell me… take care of yourself and take that 30 minmin, like, take a break…. I do find myself wanting to achieve and to be on top of things. So it’s hard for me to want to take a lunch…. I think for mental health purposes and whatnot, that wouldn’t be super favorable as far as like trying to get away for a minute and then you’re still getting those alarms.” P5

Participants shared that they will often receive messages and notifications on their personal devices through mobile applications and phone communication, at times when they are not the responding physician or APP, or when they are not the appropriate provider for a particular question.

“If someone messages me, I have that turned off because sometimes when you’re off, people will still message you and it’ll be like 3 in the morning.” P9

“I don’t use them because I’m actually, I’m too tethered to [my mobile phone]… in my practice, I’m constantly in touch with the hospital 24 h a day, seven days a week, whether I’m on call or not.” P13

The topics of personal well-being, presence, and ability to separate from work-related devices often came up when discussing smartwatches. Several participants expressed that adding a watch of similar device to the already numerous communication tools (Table 5) could be too much of a burden on workflow and personal well-being. Further, it introduced the concept of separating work devices. When developing AI tools and their user interfaces, it is important to consider the need to separate from work-related messages when the clinical team is not the currently responsible clinician. The user interface could include customization from both unit and individual levels and could include a silence feature, a sign-out feature, or the ability to easily turn notifications on and off.

### 4.3 AI usefulness across caregiver roles and experience level

Participants expressed sentiments about the *perceived usefulness* of AI technology relative to their own role and opinions on how other team members may perceive its usefulness.

“It’s funny, I think some people who’ve been doing it for a long time are not going to benefit from it because they’re not going to trust it. It might be harder for them to, like, rely on it, or fully embrace it, whereas people who kind of come in early in their career or start with it will always become used to it. And that’s just kind of like human nature.” P11

Participants suggested that more experienced team members may be less likely to use AI in a clinical context. In addition, comfort with technology appeared to be a contributing factor in hesitancy to adopt AI in the clinical setting. This could be attributed to a combination of *perceived usefulness* based on clinical experience and *perceived ease of use* based on comfort with technology use.

“As a new nurse, I think that I would definitely benefit from something like that. But I also think that it’s always good to evolve with changing things, so as an experienced nurse, I think it’s good to be able to take new things as things change.” P10

P3 is an experienced nurse and indicated they did not believe the technology would benefit them, given their experience, but thought new people would benefit from AI support. P3 also mentioned that “technology can be frustrating” when discussing different technologies in the clinical space. P2 shared that their comfort level with new technology “depends on the technology,” if they are used to it, “it is great,” but it ”takes a while to learn something new.”

This reinforces the need for a rigorous onboarding plan, a UI that aligns with current practices, and an iterative co-design process.

Additional considerations not already discussed may influence the *perceived ease of use*, including ease of accessing the data with an easy-to-use login system and accessibility within the current workflow. For instance, charge nurses need easy access to an overview of all patients. Meanwhile, bedside nurses may be monitoring patients in different rooms and need remote access to patient status information.

Charge nurses are “like air traffic control.” P4

“Let’s say I had two patients… and you’re in your other patient’s room… but you want to still see how that guy’s blood pressure is doing. A nice thing that you can do is pull up a small little mini-screen within your larger monitor screen to still be able to see what is going on in… your other patient’s room.” P5

## 5 Discussion

The CT-ICU workforce is a highly functioning team caring for high-acuity patients after cardiothoracic surgery. The findings of this field study highlight important factors to consider when implementing predictive analytic algorithms and other technologies in this complex, high-stress environment. The general sentiment of measured optimism of participants about the use of AI in the CT-ICU suggests that careful attention to the needs, concerns, and potential barriers to implementation of the clinical team, through an iterative design process with clinical team feedback, is important to build trust and understanding of AI systems. Appropriately calibrating user trust in any AI system is critical and will ultimately minimize risks of both over-trust/over-reliance and total mistrust, leading to abandonment (24, 44–46).

### 5.1 Deductive framework mapping to the technology acceptance model

The Technology Acceptance Model (TAM) is one of the most widely accepted theories on technology acceptance (41). TAM theorizes that technology acceptance is influenced by two constructs: *perceived usefulness* and *perceived ease of use* (41). In consideration of the adoption of AI technology in the CT-ICU setting, thematic findings were evaluated through the TAM lens, revealing factors that drive or block acceptance of AI technology.

Looking to the findings, added clinical value is a clear driver of *perceived usefulness*: if the system provides reliable, actionable insights not already known to the care team, there is a compelling case for adoption, despite implementation challenges. By contrast, added burdens via workflow, alarm fatigue, documentation, and technostress are strong inhibitors to *ease of use* that need to be considered and minimized for adoption success.

Each key insight from our study was mapped using deductive framework mapping (40) to one or both of the TAM constructs (41). Key findings and insights are mapped in Section 5.2 as *perceived usefulness* 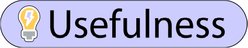, *perceived ease of use* 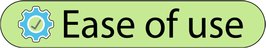, or both 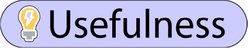 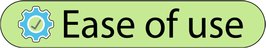. Thus, our work bridges the gap between research innovation and real-world clinical implementation and adoption of new technologies.

### 5.2 Summary of key findings and insights

Here, we identify key findings and design implications derived from our observations. Figure 2 outlines the most important design insights with sample participant quotes to add additional context.

**Ensure added clinical value.** 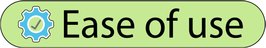 Complexity and volume of information dealt with on a minute-by-minute basis by the CT-ICU staff means that new pieces of information (such as algorithm-derived risk indicators) are treated with some skepticism and are unlikely to be integrated into clinical workflows unless they provide significant clinical decision-making value. This means that predictive algorithms not only need to produce accurate predictions, but also need to do so in a timely manner, be useful for meaningful intervention, and they must make predictions that providers could not easily have made themselves based on clinical experience. In other words, adoption of AI depends on the added value beyond what is already known.

**Engage the clinical team in a co-design process.** 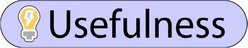 Full engagement in the design process is important to identify domain-specific information that may not be apparent to a non-clinician. Additionally, it will serve to gain care team buy-in, improve trust in the system, and ensure a strong fit with clinical workflows. This finding aligns with prior literature in related settings, with a movement toward early engagement with stakeholders in a co-design process and reinforces existing best practices for clinical technology design (16, 46–48).

**Determine the purpose of the system.** 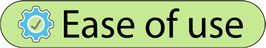 AI systems can have different purposes and functions, including diagnostic assistance or clinical management. Sutton et al. (49) classified the various categories of AI use in a clinical setting, including patient safety (error reduction), clinical management (treatment guideline support), cost containment, administrative function, diagnostic support, and patient-facing support (25). Additionally, AI technology can have differing levels of autonomy. Although actionable insights are important, maintaining the clinician as the decision-maker in a **human-in-the-loop** model is necessary in the CT-ICU setting and, arguably, in all medical settings.

**Establish workflow-aligned risk thresholds**. 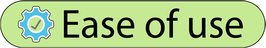 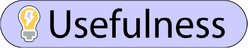 Workflow-aligned risk thresholds and alarm thresholds can influence perceived *added clinical value*. Risk thresholds, or risk level to the patient, must be determined at levels that are meaningful to patient care and at levels that would prompt actionable insights. This may be considered in the context of current clinical practice. Engagement of the clinical team in determining thresholds is essential. Additional validation of thresholds may be necessary through clinical testing and/or clinical trials.

**Establish workflow-aligned alert or alarm levels.** 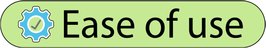 Link predictive-algorithm risk scores to alarms while minimizing alarm fatigue. Alarm fatigue, a critical challenge in intensive care environments, is defined as an excess of alarms leading to alarm desensitization (50–54). Alarm fatigue is more than a burden to workflow; it is a serious safety issue and can lead to critical alerts or alarms being inadvertently ignored and delay in the delivery of critical care. Risk scores that are calculated passively in the background and simply displayed on a device as a time-varying value are unlikely to attract needed attention in this busy and complex environment. Thus, more salient alarms or alerts will be necessary if the risk is serious and imminent. Yet conversely, alarms generated from risk score thresholds could add to the already burdensome alarm fatigue in CT-ICUs. Navigating this delicate balance may require careful choices in design, such as well-chosen threshold values for triggering alarm states,the ability to customize alarm presentation, and the use of silent alarms such as device notifications or visual displays.

**Ensure accurate and timely data are used.** 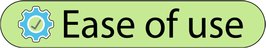 Technological malfunctions and human factors can contribute to erroneous data, thereby adversely affecting AI predictions. Staff expressed that they would need confidence in the validity of the underlying data as a prerequisite to building trust in a predictive AI system. The CT-ICU team is trained to detect technological errors and investigate data in the context of the overall clinical situation. Current practices allow team members to validate data when a concern arises. **Including a data validation process** in the system and educating the clinical team on data validity during **rigorous onboarding** must be considered.

**Ensure relevant AI training data are used.** 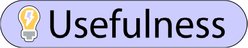 Unique patient populations can impact the performance of machine learning models and reduce trust in AI technology. Tailoring training datasets to local or comparable populations and involvement of healthcare providers in the iterative design process may help address this issue.

**Display temporal trends**. 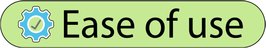 Patient data trends over time offer valuable information for the clinician’s medical assessment of a patient. Displaying temporal data as **detail-on-demand** avoids cluttering the primary view, while still allowing the clinical team to investigate trends.

**Display top AI features/reasons for each prediction.** 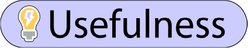 Transparency in predictions fosters trust and provides additional information for the clinician’s medical assessment of the patient. Displaying the machine learning features as **detail-on-demand** avoids cluttering the primary view. Transparency was a key factor in trust in AI systems, and ensuring transparency in predictions improves trust and usability.

**Reveal uncertainty in predicted scores and actions clinical staff can take to reduce it.** 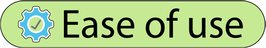 Uncertainty is an important consideration in the development of tools for patient care. Two types of uncertainty need to be considered in the clinical context: uncertainty in the prediction due to missing information or inherent uncertainty of the prediction. Knowing, for example, that information in the patient record is several hours old, or that critical test results are missing, might prompt action to update the chart or order specific clinical tests. Such features have been explored in the context of sepsis prediction (55) and could be expanded to other predictive analytics systems.

**Accessibility in current workflows.** 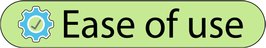 While there are workflow commonalities among the clinical team, there are also variations based on roles and individual comfort level with technology. Ease of data access and incorporation into the current workflow, without excessive burden, are key factors in technology adoption. Data access points for AI in a CT-ICU may vary slightly by institution. Nonetheless, flexibility in presenting information across multiple locations is likely important for addressing the needs of all roles in the care team. This may include an always-visible bedside screen set to the local patient view by default; a view at the nurses’ station set to show information for the whole unit, with the ability to toggle to individual patient information; the ability to access the information through the computer on wheels (COWs) or other shared workstations; and the ability to access on mobile devices. This suggests that a dedicated, readily accessible screen, or the integration of the information into primary patient views in the electronic record system, would be important for ensuring that AI predictions are seen without requiring excessive acknowledgments. In addition, **customization at the individual and unit levels**, with filtering for select patients through an easy-to-use interface, will support the different roles on the team.

**Minimize click fatigue.** 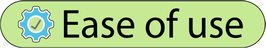 While these concepts may seem obvious from basic user experience principles, we believe they are worth calling out specifically for work in critical care domains. Click fatigue is similar to alarm fatigue but is related to a high number of alerts and clicks required in electronic medical records (56). In the critical care settings, click fatigue an lead to important notices not receiving the attention needed or being ignored. The high-stakes, complex, and stressful working conditions in a CT-ICU make minimizing burden, even the occasional extra click, particularly important for patient safety, caregiver well-being, and the likelihood of technology adoption. The theme of personal well-being was brought up by several participants. Provider burnout is a serious issue in intensive care settings and has been linked to patient outcomes (57, 58).

**Integration with current documentation systems and processes.** 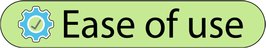 Concerns about the increased **documentation burden** with the introduction of the AI algorithm were prevalent. Notably, electronic medical records have been associated with an increased documentation burden for healthcare providers (59). This suggests value in integrating predictive algorithms into electronic medical systems so that AI predictions and evolving predicted risk scores can be automatically documented in the patient record.

**Minimize the technostress associated with new technology.** 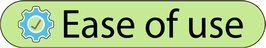 Current technology burden in the CT-ICU is high. *Technostress* is the additional stress experienced with increasing technology burden (60, 61, 69). Introducing new AI technology has the potential to increase *technostress* among the clinical team. Technostress may be higher when users have a lower level of comfort with technology or a high burden of current technology. A systematic review by Rasool et al. (61) found that training in using complex technology systems can reduce the burden. Involvement of the clinical team in **co-design**, following **conventional and domain-specific user interface heuristics** in the design, and engaging a **rigorous onboarding** for new technologies introduced in the CT-ICU can reduce the burden of learning to use new technology and are strategies to reduce the cognitive load and technostress associated with learning new AI technologies and improve adoption. Consider the need to separate from work-related messages when the clinical team member is not currently the clinician responsible. The user interface could include **customization at the unit and individual levels** and may include a **alert silence feature**, a **sign-out feature**, or the **ability to turn off notifications**. Enabling separation from personal devices may reduce technostress and promote personal wellbeing.

**Engage in evidence-based practices to develop guidelines or protocols.** 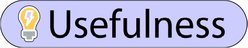 The development of clinical guidelines or nursing protocols to accompany machine-learning predictions would benefit from clinical trials demonstrating improved outcomes over the current standard of care. Thus, requiring an iterative design-and-implementation plan. To avoid becoming *noise*, clinical trials must demonstrate added clinical value to patients and care teams through improved outcomes or workflow. Medico-legal implications are complex, beyond the scope of this paper, and must be considered when implementing an AI system in the clinical setting.

### 5.3 Trusted AI for clinical teams (TACT) framework

As a synthesis of our findings, we introduce TACT, an empirically grounded, suggested framework for the development and design of AI technology with a focus on trust and adoption by clinical teams. Figure 3 outlines the proposed framework and important insights for building trust in an AI system with sample quotes for context. The framework includes five recommended stages with eight steps during those stages and seventeen key insights into the process.

**Figure 3:**
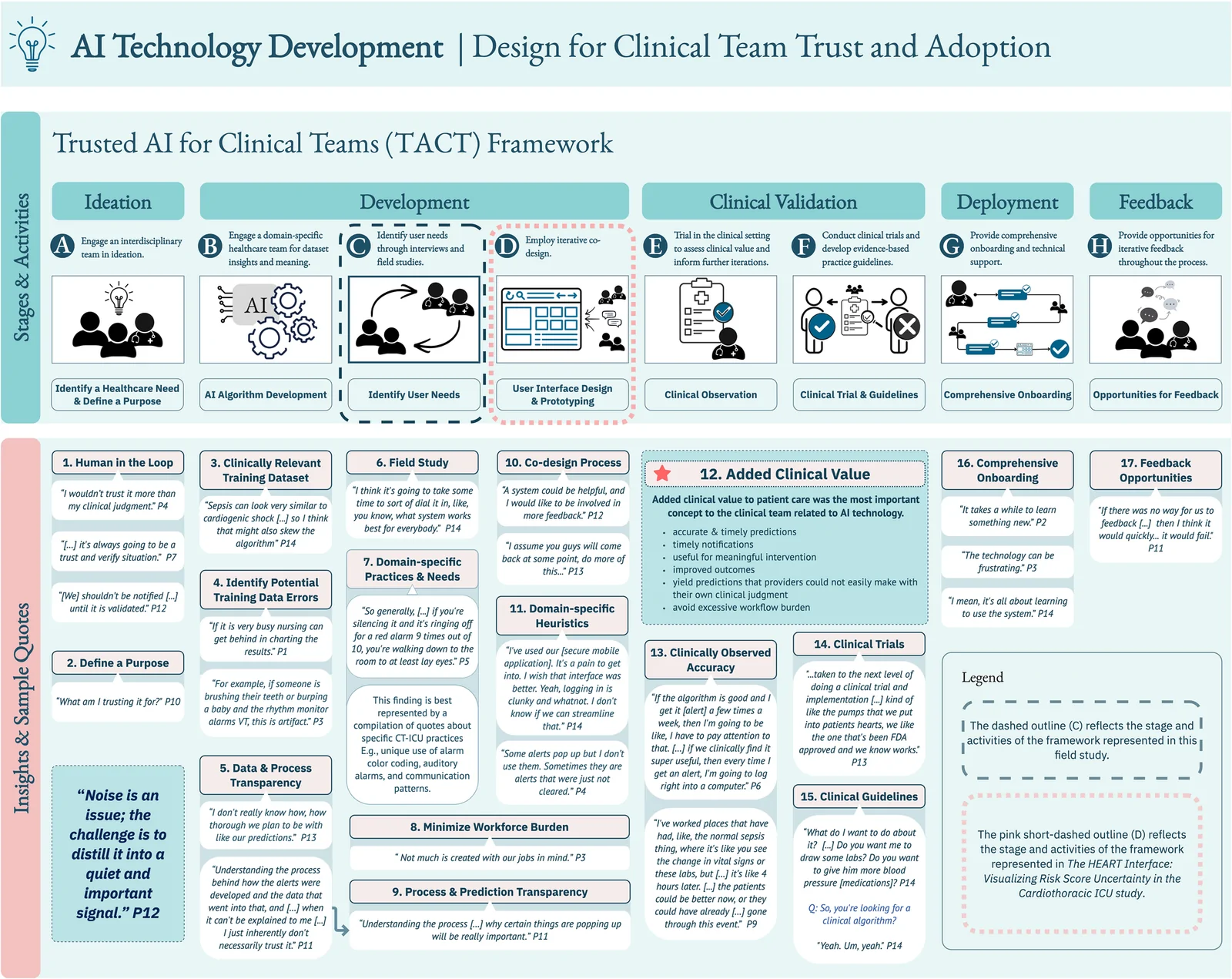
Trusted AI for Clinical Teams (TACT) Framework is a suggested framework for AI technology design and development with a focus on clinical team trust and adoption. The framework includes five development stages (Ideation, Development, Clinical Validation, Deployment, and Feedback) and eight aligned activities [columns **(A)** through **(H)**]. The lower box (pink) includes 17 activity-aligned insights from our field study, focused on improved trust and adoption. During **ideation**, **(A)** engage an interdisciplinary team to identify a healthcare need and define a purpose of the AI technology. During **development**, **(B)** engage a domain-specific healthcare team for dataset insights and meaning to support AI algorithm development, **(C)** conduct a field study with domain-experts to identify user needs, and **(D)** engage the clinical team in an iterative co-design process for interface design and prototyping. To establish **clinical validation**, **(E)** conduct trials in a clinical setting for observation accuracy and **(F)** conduct clinical trials and develop clinical guidelines. During initial **deployment**, **(G)** provide comprehensive onboarding and technical support. Lastly, **(H)** provide opportunities for iterative **feedback** throughout the process. It should be noted that at any point earlier stages in the AI development and design process may need to be revisited. Development aligned activity in column **(C)** represents the process in this field study and the development activities in column **(D)** were reflected in the *The HEART Interface: Visualizing Risk Score Uncertainty in the Cardiothoracic ICU* study (62).

The initial stage is **ideation**, with the participation of an interdisciplinary team to identify a healthcare need and define a purpose for the AI technology. Key considerations during this step include the human-in-the-loop model as a primary model and clearly defining the purpose and intent of the proposed AI technology.

During **development**, there are three key activities, including (1) engaging a domain-specific healthcare team to gain insight and meaning to the data for AI algorithm development, (2) conducting field studies with domain-experts to identify user needs, and (3) engaging the clinical team in an iterative co-design process for interface design and prototyping. Additional key insights for AI algorithm development are ensuring that the training data for machine learning are clinically relevant for the intended population, identifying potential errors in the training data, and ensuring transparency in the data and in the process to build trust. Field study goals include identifying domain-specific practices and needs, identifying ways to minimize workforce burden, and ensuring that there is transparency throughout the process. The study presented in this paper represents the field study stage of development in the TACT framework. During user interface design, the needs identified in the field study are used for initial prototyping, and then the clinical team is engaged in an iterative co-design process. The findings of this field study were used to inform the successful co-design process described by Nourani et al. (62) and represent the initial aspects of the co-design stage in the TACT framework (Figure 3). While the findings of this study informed the design goals and initial design used in the study by Nourani et al. (62), no results or quotes from the current study were reported in the other paper. The steps taken in development are key to ensuring added clinical value, minimizing workforce burden, and using domain-specific heuristics.

To establish **clinical validation**, trials in a clinical setting support observation of prediction accuracy in the clinical setting, and conduct clinical trials to compare outcomes to the standard of care demonstrate effectiveness and build trust. Further, developing evidence-based clinical guidelines establishes actionable insights and overall added clinical value to AI technology. In the **deployment** stage, a comprehensive onboarding plan and opportunities for ongoing **iterative feedback** throughout the process are essential to further build trust in AI technology and adoption by the clinical team.

Previous work on AI development and implementation frameworks has focused on evaluation of AI technology for implementation in a healthcare system (33, 35, 63), the activities needed for successful implementation (64), and responsible and ethical AI use, and regulatory considerations (65–68). Table 6 provides a high level comparison of TACT to three frameworks. Although there is overlap, our suggested TACT framework offers a unique focus on design and development for clinical team trust and adoption and offers a deeper focus human factors and user interface design.

**Table 6:** Comparison of TACT to selected AI evaluation and implementation frameworks.

Framework Comparison	Trusted AI for clinical teams (TACT)	Framework for appropriate implementation and review of artificial intelligence (FAIR-AI) (33)	British Standard BS30440: validation framework for the use of AI in healthcare (34)	Translational Evaluation of Healthcare AI (TEHAI) (35)
Description	Preliminary framework for AI development focused on clinical trust and adoption.	Guide for pre- and post-implementation evaluation of AI solutions in health systems.	Framework for evidence needed to validate healthcare AI across the product life cycle.	Evaluation of clinical AI in research and working clinical systems.
Target audience	AI Technology Developers	Health Systems	AI Technology Developers	Researchers and Health Systems; AI Developers & Vendors
Purpose	Development of AI technology for clinical team trust & adoption.	Evaluation of AI technology for adoption in a health system.	Evaluate and validate AI technology across the life cycle.	Evaluate AI technology product in healthcare systems.
Methods	Qualitative field study in the CT-ICU with synthesis of findings.	Narrative review, stakeholder interviews, and expert consensus.	Literature review and consultation with subject matter experts.	Critical review of existing evaluation frameworks and gaps.
Constructs	5 Stages: Ideation, Development, Clinical Validation, Deployment, Feedback	3 Core Components: Triage, Categorize Risk, Safe AI Plan	5 Phases: Inception, Development, Validation, Deployment, Monitoring	3 Components: Capability, Adoption, Utility
Insights	5 stages, 8 activities, 17 insights focused on clinical trust. Additional system and user interface design insights provided.	3-construct approach with best practices and screening and review question tools for AI technology risk assessment for healthcare institution implementation.	5 phases, 18 assessment criteria intended to evaluate safety, quality, and performance of healthcare AI products.	3-component, 15-subcomponent rubric with weighted, traffic-light scoring.

Description, target audience, purpose, methods, and insights for four frameworks relevant to clinical AI systems.

Framework comparisons include a brief description of the framework (Description), the intended audience (Target Audience), the framework’s stated purpose (Purpose), the methodology used to develop the framework (Methods), the high level constructs of the framework (Constructs), and a summary of key insights generated by or associated with the framework (Insights).

While the framework was developed for the CT-ICU, the framework may be applicable across healthcare domains.

### 5.4 Limitations and ongoing work

There were several limitations to the study and future work is needed to examine AI implementation in clinical settings.

**Single institution and small sample size.** The in-depth, on-site nature of our field study meant that it was a single-institution study with a small sample size of 14 participants. Future studies should aim to compare our findings with those from other CT-ICUs and critical care settings. Further research will be needed to determine the extent to which our findings in the CT-ICU extend beyond the specific setting we studied.

**Limitations of audio transcription and prohibited video and photography.** Video and photography were prohibited, and audio recording directly in the CT-ICU was not possible due to patient privacy. Additionally, one participant did not wish to be recorded. Interviews were recorded in written notes (5 of 14 interviews) for observational interviews conducted directly in the CT-ICU and when a participant declined audio recording. Observational interviews had the advantage of revealing work directly in context; however, some data or nuance may have been missed in these non-recorded interviews.

**Participants were not inclusive of all healthcare roles.** While key team members were identified based on their likely roles in AI and predictive analytics, the sample does not represent the entire team. Gatekeeper selection effects are possible due to the role of CT-ICU leadership in identifying interview candidates. Further work evaluating needs and barriers related to AI could include pharmacists, physical therapists, and speech-language pathologists, among others.

**Insights not grounded by a specific prototype.** Participants in this study were not shown a prototype or sample interface. It is possible that presentation of a prototype would alter participant perceptions. Results from this study are directly informing the design of a predictive AI algorithm and a clinical interface, which we are developing in a participatory design process with our CT-ICU collaborators. To date, the insights from this study have been instrumental in establishing a foundation for design and informing design decisions. Initial co-design work has focused on visual representations of predicted AI outcomes and their uncertainty due to missing data (62), an important design consideration that was directly identified through this fieldwork. As our work progresses, additional design experimentation will be needed, as well as formal acceptance testing within the CT-ICU.

**Limitations on clinical guidelines.** Participants wanted clinical guidelines indicating how to respond when an adverse outcome is predicted; however, the medico-legal implications are complex and must be considered when implementing an AI system in the clinical setting. Following typical practices to develop evidence-based medicine protocols is important, but the depth of medico-legal considerations is beyond the scope of this study.

**A single researcher conducted the coding and thematic analysis.** A single coder with clinical domain expertise conducted the primary coding and thematic analysis. Although this approach is in alignment with the principles of reflexive thematic analysis described by Braun and Clarke (39) and it offers deep knowledge of the clinical context for interpretation, we recognize this could introduce bias. To mitigate domain bias and ensure an interdisciplinary approach was leveraged, the codebook was regularly reviewed and refined with a senior author with human-computer and human-centered AI expertise.

**Extended duration from data collection to manuscript submission.** The data for this study were collected in November 2023, and the manuscript was submitted in July 2026. Changes in participant perceptions during that time are possible. Although there have been advancements in specific AI technologies in that time, clinical trust, acceptance, and adoption of AI technology remain in their infancy and we believe the findings remain relevant to AI technology in the CT-ICU and critical care space. Additionally, our findings were situated in the current literature.

**Preliminary nature of the TACT framework and need for validation.** TACT is a preliminary framework developed as an outcome of this single site study, with potentially broad applicability to healthcare technology design. Future work will be needed to validate this framework across a variety of healthcare settings.

## 6 Conclusion

The CT-ICU healthcare team is collaborative and high-functioning. The findings of this study inform key implementation considerations when developing a predictive analytic algorithm and designing a user interface for the clinical team in the CT-ICU. Key considerations regarding trust in AI in a clinical setting were identified. Key themes of (1) the system must have *added clinical value* to the clinical team and patient, (2) *trust in the AI system* through co-design processes, transparency in the system, and consistent prediction accuracy, and lastly (3) *addressing workflow factors* by optimizing integration into current workflow with rigorous onboarding and minimizing alarm fatigue, charting burden, click burden, and technology burden, were important to the clinical team and are important considerations for team wellness and adoption success. Furthermore, for system customization, developing an intuitive interface and maintaining the hierarchy of alarms and communication will be essential. Based on our findings, we introduced TACT, a preliminary framework for clinical technology design. These findings offer potential to bridge research to clinical implementation of AI tools in critical care settings.

## Data Availability

The datasets presented in this article are not readily available because of participant confidentiality. Requests to access the datasets should be directed to c.barry@northeastern.edu.
