# Supplementary material for: Needs and barriers of healthcare teams to implement real-time AI & predictive analytics in cardiothoracic ICU: a field study
Source: Front Digit Health. 2026 Oct 30;8:1943663. doi: 10.3389/fdgth.2026.1943663 (PMC13759844; doi:10.3389/fdgth.2026.1943663)
Supplement: Supplementary file 1 [file Datasheet1.pdf]

Supplementary Material

1 COREQ CHECKLIST

Consolidated Criteria for Reporting Qualitative Research (COREQ) Checklist

Adapted from: Tong A, Sainsbury P, Craig J. Consolidated criteria for reporting qualitative research (COREQ): a 32-item checklist for interviews and focus groups. Int J Qual Health Care. 2007;19(6):349–357. Tong et al. (2007)

No.	Item	Guide Question / Description	Reported
**Domain 1: Research team and reflexivity**			
Personal characteristics			
1	Interviewer/facilitator	Which author(s) conducted the interview or focus group?	sec. 3.4 Observations and Interviews
2	Credentials	What were the researcher's credentials, e.g., PhD, MD?	sec. 3.2 Positionality Statement
3	Occupation	What was their occupation at the time of the study?	sec. 3.2 Positionality Statement
4	Gender	Was the researcher male or female?	not reported
5	Experience and training	What experience or training did the researcher have?	sec. 3.2 Positionality Statement
Relationship with participants			
6	Relationship established	Was a relationship established prior to study commencement?	sec. 3.3 Participants
7	Participant knowledge of the interviewer	What did participants know about the researcher (e.g., personal goals, reasons for doing the research)?	sec. 3.4 Observations and Interviews; & Table 1
8	Interviewer characteristics	What characteristics were reported about the interviewer/facilitator (e.g., bias, assumptions, reasons/interests in the research topic)?	sec. 3.2 Positionality Statement
**Domain 2: Study design**			
Theoretical framework			
9	Methodological orientation and theory	What methodological orientation was stated to underpin the study (e.g., grounded theory, discourse analysis, ethnography, phenomenology, content analysis)?	sec. 3.5 Data Analysis

Continued on next page

No.	Item	Guide Question / Description	Reported
Participant selection			
10	Sampling	How were participants selected (e.g., purposive, convenience, consecutive, snowball)?	sec. 3.3 Participants
11	Method of approach	How were participants approached (e.g., face-to-face, telephone, mail, email)?	sec. 3.3 Participants
12	Sample size	How many participants were in the study?	sec. 3.3 Participants
13	Non-participation	How many people refused to participate or dropped out? Reasons?	sec. 3.3 Participants
Setting			
14	Setting of data collection	Where was the data collected (e.g., home, clinic, workplace)?	sec. 3.4 Observations and Interviews
15	Presence of non-participants	Was anyone else present besides the participants and researchers?	Table 1
16	Description of sample	What are the important characteristics of the sample (e.g., demographics, date)?	sec. 3.3 Participants; & Table 1 & 2
Data collection			
17	Interview guide	Were questions, prompts, guides provided by the authors? Was it pilot tested?	sec. 3.4 Observations and Interviews; & supplemental materials
18	Repeat interviews	Were repeat interviews carried out? If yes, how many?	sec. 3.3 Participants
19	Audio/visual recording	Did the research use audio or visual recording to collect the data?	sec. 3.3 Participants
20	Field notes	Were field notes made during and/or after the interview or focus group?	sec. 3.4 Observations and Interviews
21	Duration	What was the duration of the interviews or focus group?	p. 5; sec. 3.4
22	Data saturation	Was data saturation discussed?	depth reported not saturation[a]; sec 3.5 Data Analysis
23	Transcripts returned	Were transcripts returned to participants for comment and/or correction?	No

Continued on next page

No.	Item	Guide Question / Description	Reported
**Domain 3: Analysis and findings**			
Data analysis			
24	Number of data coders	How many data coders coded the data?	sec. 3.5 Data Analysis
25	Description of the coding tree	Did authors provide a description of the coding tree?	sec. 3.5 Data Analysis
26	Derivation of themes	Were themes identified in advance or derived from the data?	sec. 3.5 Data Analysis
27	Software	What software, if applicable, was used to manage the data?	sec. 3.5 Data Analysis
28	Participant checking	Did participants provide feedback on the findings?	No
Reporting			
29	Quotations presented	Were participant quotations presented to illustrate the themes/findings? Was each quotation identified (e.g., participant number)?	sec. 4 Findings; & Tables 1, 2, 3
30	Data and findings consistent	Was there consistency between the data presented and the findings?	sec. 4 Findings; & Figures 1, 2, 3
31	Clarity of major themes	Were major themes clearly presented in the findings?	sec. 4 Findings; sec. 5 Discussion; & Figures 1, 2, 3
32	Clarity of minor themes	Is there a description of diverse cases or discussion of minor themes?	sec. 4 Findings; sec. 5 Discussion; & Figures 1, 2, 3

[a] Depth prioritized over saturation in alignment with Braun and Clarke Thematic Analysis Braun and Clarke (2006, 2019, 2021).

REFERENCES

Tong A, Sainsbury P, Craig J. Consolidated criteria for reporting qualitative research (COREQ): a 32-item checklist for interviews and focus groups. *International Journal for Quality in Health Care* **19** (2007) 349–357. doi:10.1093/intqhc/mzm042.

Braun V, Clarke V. Using thematic analysis in psychology. *Qualitative Research in Psychology* **3** (2006) 77–101. doi:10.1191/1478088706qp063oa.

Braun V, Clarke V. Reflecting on reflexive thematic analysis. *Qualitative Research in Sport, Exercise and Health* **11** (2019) 589–597. doi:10.1080/2159676X.2019.1628806.

Braun V, Clarke V. To saturate or not to saturate? Questioning data saturation as a useful concept for thematic analysis and sample-size rationales. *Qualitative Research in Sport, Exercise and Health* **13** (2021) 201–216. doi:10.1080/2159676X.2019.1704846.
