# Supplementary material for: Needs and barriers of healthcare teams to implement real-time AI & predictive analytics in cardiothoracic ICU: a field study
Source: Front Digit Health. 2026 Oct 30;8:1943663. doi: 10.3389/fdgth.2026.1943663 (PMC13759844; doi:10.3389/fdgth.2026.1943663)
Supplement: Supplementary file 2 [file Datasheet2.pdf]

Supplemental Materials

Needs and Barriers of Healthcare Teams to Implement Real-Time AI & Predictive Analytics in Cardiothoracic ICU: A Field Study

Codebook

Name	Description	Sources	References
**RQ1 Current Practices**	**Aggregate of Current Practices**<br>**Codes in this group are expressed as current practices.**	**14**	**1071**
**Alarm Customize or Silence**	Alarm or alert customization or silence	8	33
**Alarm Parameter Customization**	Customization of alarms or alerts	7	20
**Alarm Silence**	Silence feature mentioned	4	10
**Alarms, Alerts, Monitors, Tools**	Alarms, alerts, monitors, or tools to receive notifications or communication	14	729
**Alarms and Alerts**	Alarms or alerts mentioned	13	361
**Alert & Alarm Hierarchy**	Current practice, mentioned notification, alert, or alarm	12	131
**Critical (Red) Alarm**	Critical or red alerts or alarms	11	54
**Intermediate (Yellow) Alarm**	Intermediate or yellow alerts or alarms	8	19
**Low (Blue or White) Alarm or Alert**	Low risk, blue, or white alert or alarms	7	12
**Auditory Alarm**	Mentioned auditory alarms	11	59
**Call bell**	Current practice, mention call bell use	1	2
**Chart Alert**	Mentioned an alert in the chart, includes passive or pop-up alerts	8	31
**False Alarm**	Mentioned alerts that are not	11	32
**Next action alert**	Alerts that give instruction on next action to take	1	6
**Patient environment disruption**	Mentioned noise disruption impacting patients	3	5
**Phone**	Mentioned phone as current method to receive alarms and alert, include mobile apps, phone calls, or text messages	4	12
**Response to alarm**	Description of how responding to an alert or alarm	9	37

Visual Alarm	Mention of a visual alert or alarm.	6	20
Communication Tools	Any communication tool discussed.	12	148
Direct conversation	Current communication, direct conversation	7	14
Email	Email or email service mentioned	1	2
EMR Chat	EMR chart communication mentioned	8	20
MS Teams	MS Teams or MS Teams chat mentioned	5	13
One Call	One call communication tool	1	1
Overhead Announcement	Overhead announcements mentioned	1	1
Pager	Pager mentioned	8	30
Phone	Phone including mobile app, phone call, and text message as a communication method.	11	43
Smartwatch	Smartwatch mentioned including al	3	6
Tools Hierarchy	Mentioned order of tools used or use related to patient condition	3	7
Patient care equipment	Tools for patient care	5	10
Patient Data Tracking	Tools for tracking patient data	14	130
EMR Chart	Mentioned EMR views,	14	99
Chart Views	Specific Views and customization	8	37
Sepsis Prediction in EMR	Sepsis	4	8
PACs - Radiograph Viewing	Radiology system to view radiographs	2	2
Paper & Other	Methods that are not in the Epic Chart	6	17
Electronic ID System	Scanning System to scan patient ID and medications or labs; used to identify and log patient medications lab draws, etc.	5	7
Patient Monitors	A monitor or screen with patient data	11	33
Independent Monitors	Monitors that are independent and do not communicate with Epic; require team member entry of data.	5	7
Integrated Monitors	Integrated with patient chart	2	4
Phillips Monitor	The monitoring and alert/alarm system through the unit, monitors heart rhythm, vital signs, etc.	9	18
Technical Issues	Technical issues	9	46
Erroneous Data	Patient data that is erroneous for any reason	8	36
Technology Malfunction	Technology malfunctions	3	5
Team & Responsibility	Aggregate team responsibilities and practices	14	299
Clinical judgement	Mention use of clinical judgement or describe clinical judgement	12	22
Demeanor	Mention team or personal member demeanor	1	1
Calm Demeanor	Mention calm demeanor	1	1
Personal Wellbeing	This refers to personal wellbeing, personal work life boundaries, or separation from work devices	8	20
Responsibilities	Aggregate responsibilities	11	74
Bed Management	Management of where patients go or which rooms or beds	1	3
Charting	Documentation in the electronic medical record	9	49
Charting Burden	Mentioned charting burden	8	39

Decrease Charting Burden	Mentioned things that decrease charting burden	6	10
Increase Charting Burden	Mentioned things that increase charting burden	5	10
Direct patient care	Mentioned direct patient care explicitly; did not include if described patient care	3	6
Rounds	The process of rounding on patients to discuss plans and patient care.	1	2
Staffing Unit (Nursing)	Assigning nursing staff to a patient on the unit	1	2
Team approach	Team approach to patient care	3	9
Team Communication	Aggregate of team communication	10	29
Communication Hierarchy	Ranking of importance of communication, methods of communication	8	14
Preferences & Ranking	Communication preferences	6	7
Team Roles	Aggregate team roles	12	131
Role Hierarchy	Mentioned role or communication ladder or chain	2	4
Team Members	Aggregate of team member roles	12	125
APP	Mentioned the role indicated	7	18
Nurse Practitioner (NP)	Mentioned the role indicated	1	2
Physician Assistant (PA)	Mentioned the role indicated	1	1
Physician	Mentioned the role indicated	5	17
Cardiovascular Critical Care (CVCC)	Mentioned the role indicated	4	7
Consultants	Mentioned the role indicated	1	1
Cardiologist	Mentioned the role indicated	1	1
Surgeon	Mentioned the role indicated	3	4
Role Nurse	Mentioned the role indicated	11	88
Experienced Nurse	Mentioned the role indicated	8	17
Newer Nurse	Mentioned the role indicated	7	20
Role Charge Nurse	Mentioned the role indicated	3	13
Staff Nurse	Mentioned the role indicated	7	14
Support Team Members	Mentioned the role indicated	1	2
ECMO Specialist	Mentioned the role indicated	1	1
Respiratory Therapist	Mentioned the role indicated	1	1
Workforce changes and needs	Changes in workforce	8	21
New in Role	Mentioned new in role	2	3
Newer Nurse	Mentioned new in nursing (defined as less than 3 years)	6	11
Unique Practices	Practices unique to the setting/location	4	9
**RQ2 Attitudes Toward AI**	**Aggregate Attitudes Toward AI**<br>**Codes in this group are expressed as negative or positive sentiment about AI technology in the CT-ICU.**	**9**	**34**

Sentiment - AI	Aggregate sentiment about AI	9	34
Negative Sentiment AI	Expressed negative sentiment about AI	5	11
Positive Sentiment AI	Expressed positive sentiment about AI	9	23
**RQ3 Barriers**	**Aggregate of Barriers & Trust**<br>**Codes in this group are expressed or described barriers to AI technology.**	**14**	**470**
Adoption of New Tech	Mentioned opinions about technology usefulness or desire to use	8	12
Awareness & Access	Team awareness and ease of access to the predictive algorithm and dashboard.	2	2
Burden	Mentioned a concern about a or current workflow burden	14	127
Alarm & Alert Fatigue	Alert or alarm fatigue, false alarms, or alarm burden mentioned or described	14	66
Alarm Fatigue	Alarm fatigue, false alarms, or alarm burden mentioned or described	12	48
Alert to Things Already Known	Alert or alarm to something already known by clinical team	2	4
Chart alert burden	Chart alert burden mentioned or described	7	10
Systems Fatigue	Mentioned or described numerous systems to learn and use, or learning new systems	8	31
No. of Systems	Speaks to the number of systems required for patient care and communication.	6	9
Task Burden	Mentioned or described tasks that are a burden in workflow	12	26
Click Fatigue	Mentioned need to click excessively or acknowledge an alert in EMR	3	7
Documentation Burden	Mentioned need to manually document patient data	8	11
Erroneous Data	Mentioned or described data errors including human or technical	7	31
Tech Use & Comfort	Aggregate mentioned or described personal technology use or comfort level	13	124
Personal Technology Use	Mentioned or described personal technology use	13	86
AI Pin	Mentioned the listed technology listed	1	2
Apple Technology	Mentioned the listed technology listed	12	50
Apple Watch	Mentioned the listed technology listed	5	6
iPad	Mentioned the listed technology listed	4	4
iPhone	Mentioned the listed technology listed	11	18
Mac Computer	Mentioned the listed technology listed	6	6
Non-Apple Technology	Mentioned the listed technology listed	8	16
Garmin Watch	Mentioned the listed technology listed	1	1
PC & Windows Based Technology	Mentioned the listed technology listed	6	8
Smartwatch	Mentioned the listed technology listed	9	18
Apple Watch	Mentioned the listed technology listed	4	5
Garmin Watch	Mentioned the listed technology listed	1	1

Tech Comfort Level	Mentioned or described personal technology comfort level	13	30
Lower Tech Comfort	Lower comfort level	3	6
Low Technology Comfort Level	Below average	3	4
Tech Comfortable	Average or above average tech comfort	13	22
High Technology Comfort Level	Above average	7	11
Moderate Technology Comfort Level	Average	6	9
Tech malfunction	Malfunction of computer systems and interactions of computer systems	2	5
Trust in AI	Trust in predictive analytics, artificial intelligence, machine learning	13	176
Decrease Trust or Value	Aggregate things that would decrease trust or value in AI, ML, predictive analytics	12	100
Conflict w Clinician	Mentioned disagreement between AI and clinical judgement or patient condition	3	7
Inaccurate Prediction	Mentioned inaccurate predictions	6	10
Relies on Flawed Data	Relies on human input or erroneous data	6	41
Human Error	Mentioned delays or error in entry into the EMR	4	9
Technical Error	Mentioned technical data errors	3	4
Unique Patient Population	Describes conditions where the patient population is unique and may not align with general patient populations.	3	6
Unclear Rationale	Lack of understanding or transparency of the model.	5	7
Increase trust	Aggregate things that would increase trust or value in AI, ML, predictive analytics	12	58
Accurate Predictions	The predictions of the algorithm are accurate	7	15
Clinical Trial or Approved	Rigorous validation methods	4	7
Clinician Involvement	Clinician involvement in development or feedback	4	4
Data accuracy	Data accuracy mentioned	3	6
Outthink Me	Added clinical value and predictions beyond clinical judgement.	5	6
Transparency	Transparency in why prediction is made and how the algorithm works and was developed.	5	7
Purpose of System	Mentioned purpose of the system	2	3
**RQ4 Important Features**	**Aggregate of Important Features**<br>**Codes in this group were design features that were expressed or described as important in an AI system.**	**14**	**667**
AI Notifications	Aggregate of AI notification	14	241
AI Alert Methods	Alert or alarm methods	11	75
Alert Customization	Alert or alarm customization	6	12
Auditory Alert	Auditory alarm	5	13
EMR Alert	EMR chart alert including active (click to acknowledge) and passive (need to view it)	6	9

EMR Custom View	Custom view in EMR or flag showing alert - passive	1	1
Open display	AI notifications as a display	5	6
Pager	Notification via pager	2	2
Phone	Notification via phone, includes call, text, or mobile app	9	14
Separate Work Device	Mentioned need to have a device separate from work	2	6
Smartwatch	Smartwatch notification	2	5
Visual alert	Visual alert for notification	2	4
AI Notification Hierarchy	Alert or notification method relative to risk level of the prediction	11	140
Change in Risk	Alert or notification method for change in risk	2	2
Critical alert RED	Alert or notification method for red alert or high/critical risk prediction	6	19
Low alert BLUE	Alert or notification method for blue alert or low risk prediction	4	9
Moderate alert YELLOW	Alert or notification method for yellow or intermediate risk prediction	2	5
Roles To Notify Benefit	Roles to notify or would benefit most to receive notification	10	90
APPs	Mentioned the role to notify or would benefit from notification	6	15
Bedside Nurse	Mentioned the role to notify or would benefit from notification	10	28
Experienced Nurses	Mentioned the role to notify or would benefit from notification	1	1
Newer nurses	Mentioned the role to notify or would benefit from notification	2	3
Charge Nurse	Mentioned the role to notify or would benefit from notification	3	8
Doctors	Mentioned the role to notify or would benefit from notification	7	25
CVCC - Intensivist	Mentioned the role to notify or would benefit from notification	5	11
Surgeon	Mentioned the role to notify or would benefit from notification	5	12
Newer People	Mentioned the role to notify or would benefit from notification	2	2
Patient	Mentioned the role to notify or would benefit from notification	1	1
PT, OT, Other	Mentioned the role to notify or would benefit from notification	1	1
Timely alerts	Alerts that are timely to allow action	3	3
AI System Properties	Aggregate of AI system properties	14	313
Avoid in AI Design	Features to avoid in an AI, ML, predictive system.	6	10
Clinical Hands	Mentioned clinical team hands or clinical care as not being replaceable	4	8
Clinician Involvement Feedback	Clinical or healthcare provider involvement in the design and ability to provide feedback.	3	3
Important in AI Design	Features that are important to have in an AI, ML, predictive system.	13	79
EMR Chart Integration	Integration into the EMR	7	13
Silence feature	Silence or pause alerts or alarms	4	9
Transparency	Transparency in why the prediction is made and how the predictive algorithm works.	8	14
Validity	Proven and observed validity of the system	4	13
Verify Data	Ability to verify data	8	28
Predictive Value CI	Important aspects of prediction confidence or uncertainty	12	81
Added Clinical Value	Capabilities beyond clinical judgement	11	37

Timely	Mentioned clinical value being impacted by timeliness	2	2
High Predictive Value	Mentioned need for high confidence to be notified	7	14
Increase in Predictive %	Notification if a prediction confidence or risk increased	3	3
Threshold Predictive Confidence Ranks	Mentioned confidence levels, ranking, or thresholds for notifications	8	21
Training and Onboarding	Training or onboarding mentioned	5	12
User Interface	Aggregate user interface features mentioned	14	117
Customizability	Customizability mentioned	8	17
Data to See	Mentions of data to see when a prediction was made	12	41
Confidence of Prediction	Confidence level or uncertainty of the prediction	8	22
Details on demand	Mentioned seeing more details of patient data	5	8
Access to data	Mentioned data to access when viewing	4	4
Factors for Prediction	Mentioned wanting to see the why a prediction was made	4	6
Easy log in	Mentioned log in challenges or need for easy log in	4	4
Feedback	Mentioned access to give feedback	4	4
Interface unique look	Mentioned a unique interface visual	1	1
Intuitive Interface	Mentioned easy to use interface	5	5
Mobile Access	Mentioned wanting mobile access or using mobile access as a positive	6	12
Silence	The ability to pause, silence, or acknowledge the alert or alarm.	5	14
Trend Over Time	See the trend over time	5	9
What To Do About It	Clinical protocols	5	8
