# Supplementary material for: Needs and barriers of healthcare teams to implement real-time AI & predictive analytics in cardiothoracic ICU: a field study
Source: Front Digit Health. 2026 Oct 30;8:1943663. doi: 10.3389/fdgth.2026.1943663 (PMC13759844; doi:10.3389/fdgth.2026.1943663)
Supplement: Supplementary file 3 [file Datasheet3.pdf]

Supplemental Materials

Needs and Barriers of Healthcare Teams to Implement Real-Time AI & Predictive Analytics in Cardiothoracic ICU: A Field Study

Supplemental Materials Contents

Interview Guides 1
Staff Meeting Introduction Guide 2
Interview Guide 3

Interview Guides

A guide for the Staff Meeting Introduction was created to guide the initial discussion. A single interview guide was developed for all participant roles, including *nursing staff, advanced practice providers (NP/PAs)*, and physicians.

Interviews will be semi-structured. The guides provide an overall plan and aim to include all major topics, but we allow the conversation to flow freely and follow interesting leads.

We preidentified the number of people to interview for each role category and will continue until we reach sufficient data depth.

Guides

1. Staff Meeting Introduction Guide
2. Interview Guide

STAFF MEETING INTRODUCTION GUIDE

Goal: Meet the staff, introduce the team, introduce the project, and answer questions.

Introduction of the Study and Team

Thank you for allowing us to join your staff meeting to introduce ourselves and discuss the project observations and interviews.

Dr. Tory will introduce the team and then discuss the project and the goal time frame.

We will observe you working in the space, tag along and ask questions, and interview you in a separate space.

We want to understand how you work today, how you interact with alarms and patients, and what you might hope for in a real-time predictive algorithm.

1. Introduction to the project
2. Introduction to the team
3. What to expect

The specific aims are to deploy a platform to capture real-time patient data, develop a computational platform and predictive algorithm to identify CT-ICU patients at risk for adverse events, demonstrate real-time implementation of the predictive algorithm, and conduct a prospective study following patients in the CT-ICU.

We are the team supporting the development of an interface to communicate this data to you, the healthcare team. To develop an effective interface, it is important for us to best understand your workflow and needs. These interviews and observations will inform this process.

GOAL Message:

- There is no evaluation – no right or wrong answers, no assessment of your work.
- We want to better understand your workflow and processes so this can work best for you and your team.
- We are not evaluating your work in any way.

We are happy to answer any questions you may have about the project or process.

INTERVIEW GUIDE

Observation Focus Topics.

- Interactions with alerts and alarms.
- Steps taken when alerts occur.
- Communication of patient data and alerts.
- Interactions with current technology.
- Clinical decision making.

Introductions

- Introduce ourselves.
- Goal of the Study: we want to understand your workflow and the needs of the healthcare team to implement predictive analytic use in the MMC CT-ICU.
- Review privacy statement and request consent

Participant Role and Background

- Your job title, department, role – what is your role here?
- Years of experience in healthcare? Years of CT-ICU experience?
- How would you describe your gender? Age?
- Describe comfort level with technology.
- Describe your current technology use.
- What tools do you use to track patient data?
- Communicate patient data and with who?

Probing Questions

Alarms and Alerts

- What alerts and types of are you currently receiving? (where, what, about who)
- What alerts is your perception of an alert hierarchy?
- Walk us through the process when you receive an alert about a patient.
- How do you receive the alerts? What additional information do you need to evaluate?
- How would you like to receive those alerts in the future? (iPhones, smart watch, sounds, flashing lights, etc.)
- Who should receive the alert first? In the ideal future.
- What steps do you take next?
  - (possible follow up – get an understanding of any additional data they access to further understand what is going on)
- What technology do you currently use? Location? How do you access it? What does it look like?
- Can you share an example of when your current process was effective? When it wasn't effective? What could have improved?
- In an ideal world how would you like to receive alarms/alerts?

Communication

- Who are the people you communicate with about the patient?
- How is the information shared? Tracked by the team?
- Example of communication flow? Who? What? How? Where? Tools?
- Can you share an example of when your current process was effective? When it wasn't effective? What could have improved?

Current Technology

- What tools do you currently use? What information is provided?
- Walk us through the process?
- What technology is effective? What is not effective? What could be improved?

Clinical Decision Making

- How do you intuitively predict if there could be an adverse event with a patient?
- Additional information? How do you obtain the information?
- If you had a magic wand, what would you like to see in a predictive analytic? Interface? Data? How?

Trust in AI

- Would you use an algorithm that predicts adverse patient events? Why or why not?
- What concerns you about bringing a predictive algorithm into the CTICU?
- What factors do you think would increase or decrease your trust in the algorithm?

Overall Reflection

- If there was one thing you could change to make your life easier, what would it be?
- Tools? Workflow?

What additional information would you like to share with us?
